# IgE Antibodies: From Structure to Function and Clinical Translation

**DOI:** 10.3390/antib8010019

**Published:** 2019-02-22

**Authors:** Brian J. Sutton, Anna M. Davies, Heather J. Bax, Sophia N. Karagiannis

**Affiliations:** 1 King’s College London, Randall Centre for Cell and Molecular Biophysics, London SE1 1UL, UK; anna.davies@kcl.ac.uk; 2 Asthma UK Centre in Allergic Mechanisms of Asthma, London, UK; 3 King’s College London, St John’s Institute of Dermatology, London SE1 9RT, UK; heather.bax@kcl.ac.uk

**Keywords:** Immunoglobulin E, FcεRI, CD23, allostery, cancer immunotherapy, AllergoOncology, IgE effector functions, monocytes, macrophages, ADCC

## Abstract

Immunoglobulin E (IgE) antibodies are well known for their role in mediating allergic reactions, and their powerful effector functions activated through binding to Fc receptors FcεRI and FcεRII/CD23. Structural studies of IgE-Fc alone, and when bound to these receptors, surprisingly revealed not only an acutely bent Fc conformation, but also subtle allosteric communication between the two distant receptor-binding sites. The ability of IgE-Fc to undergo more extreme conformational changes emerged from structures of complexes with anti-IgE antibodies, including omalizumab, in clinical use for allergic disease; flexibility is clearly critical for IgE function, but may also be exploited by allosteric interference to inhibit IgE activity for therapeutic benefit. In contrast, the power of IgE may be harnessed to target cancer. Efforts to improve the effector functions of therapeutic antibodies for cancer have almost exclusively focussed on IgG1 and IgG4 subclasses, but IgE offers an extremely high affinity for FcεRI receptors on immune effector cells known to infiltrate solid tumours. Furthermore, while tumour-resident inhibitory Fc receptors can modulate the effector functions of IgG antibodies, no inhibitory IgE Fc receptors are known to exist. The development of tumour antigen-specific IgE antibodies may therefore provide an improved immune functional profile and enhanced anti-cancer efficacy. We describe proof-of-concept studies of IgE immunotherapies against solid tumours, including a range of in vitro and in vivo evaluations of efficacy and mechanisms of action, as well as ex vivo and in vivo safety studies. The first anti-cancer IgE antibody, MOv18, the clinical translation of which we discuss herein, has now reached clinical testing, offering great potential to direct this novel therapeutic modality against many other tumour-specific antigens. This review highlights how our understanding of IgE structure and function underpins these exciting clinical developments.

## 1. Introduction

Immunoglobulin E (IgE), named in 1968 [1,2,3], was the last of the five classes of human antibodies to be discovered, and today is commonly associated with the various manifestations of allergic disease [4]. However, its role in mammalian evolution appears to be the provision of a mechanism for defence against parasites and animal venoms [5], and in this regard it required the acquisition of a powerful effector function. It is precisely this power, and the possibility of understanding and harnessing it, that makes IgE an attractive candidate for monoclonal antibody immunotherapy against clinically important targets. IgE differs from the various sub-classes of IgG that have hitherto been the common format for therapeutic antibodies in a number of key aspects, including its domain architecture, glycosylation, conformational dynamics and, as only recently appreciated, allosteric properties [6]. In this review, we bring together our understanding of the structural and functional properties of IgE, and show how this underpins the development of IgE as a therapeutic antibody format. 

IgE’s receptor-binding activities also present unique features. There are two principal receptors, FcεRI, structurally homologous to other members of the FcγR family, and FcεRII/CD23, which unlike almost all other antibody receptors, is a member of the C-type (Ca^2+^-dependent) lectin-like superfamily [4]. FcεRI is expressed on tissue mast cells, blood basophils, airway epithelial and smooth muscle cells, intestinal epithelial cells, and various antigen-presenting cells (APCs), monocytes and macrophages [7,8,9,10,11]; the cross-linking of receptor-bound allergen-specific IgE on mast cells and basophils by allergen is the signal for cell degranulation, the release of pre-formed mediators of inflammation and an immediate hypersensitivity response that can be powerful enough to cause anaphylactic shock and even death. Not only is it is necessary to cross-link very few IgE and FcεRI molecules in this way, compared with IgG and FcγR, but the affinity of IgE for FcεRI (K_a_ ≈ 10^10^ M^−1^) is at least two orders of magnitude higher than that of IgG for any of its receptors. Thus, most IgE is already cell bound, and all that is required is contact with perhaps a minute amount of allergen to trigger a rapid reaction. In contrast, IgG generally requires the formation of immune complexes consisting of many more antibody molecules, which can then, upon contact with an effector cell, cause FcγR clustering and cell activation [12]. With its uniquely high affinity for any antibody-receptor interaction, FcεRI is often referred to as the “high-affinity” receptor for IgE.

FcεRII, or CD23 as it will be called here, is also known as the “low-affinity” receptor for IgE. While the affinity of each of its lectin-like “heads” for IgE (K_a_ ≈ 10^6^ M^−1^) is indeed much lower than that of FcεRI, the fact that the molecule is trimeric can lead to a higher avidity if more than one head can engage IgE; this will be discussed in detail later. CD23 is expressed on B cells, T cells, various APCs, gut and airway epithelial cells and a range of other cell types [13,14,15,16,17,18]. On B cells, IgE binding to CD23, the latter behaving both as a membrane protein and also as a soluble protein released from the cell surface (in trimeric or monomeric form) by endogenous or exogenous proteases, can either up- or down-regulate IgE levels [13,19,20,21]. This interplay between IgE and both membrane and soluble CD23 has been proposed to constitute a mechanism for IgE homeostasis. CD23 also transfers IgE-allergen complexes across the airway and gut epithelia and thus promotes the presentation of airborne and food allergens to the immune system [16,17,18,22].

There is a considerable body of structural data concerning the interactions between IgE-Fc and the receptors FcεRI and CD23. There is also a good understanding, if based upon rather few examples, of how IgE Fabs recognise allergens; this understanding was recently enhanced by the discovery that allergen recognition may occur not only in a classical, complementarity-determining region (CDR)-mediated manner, but also through V-region framework regions (FR) in a “superantigen-like” mode [23]. When we put these structural data together to build models of the whole IgE molecule, it is clear that there are constraints upon the disposition of the Fab arms when the Fc is receptor bound, and similarly, there may be restrictions upon the receptor-binding capability of the Fc region when IgE engages target antigens; unfortunately, we lack high-resolution structural data on the complete IgE molecule. Appreciation of these constraints and the consequences of the flexibility and dynamics of the IgE molecule as a whole, are clearly important for engineering an IgE molecule for immunotherapy that combines the desired antigen-binding and receptor-mediated activities.

## 2. The Structure of IgE

**Figure 1:**
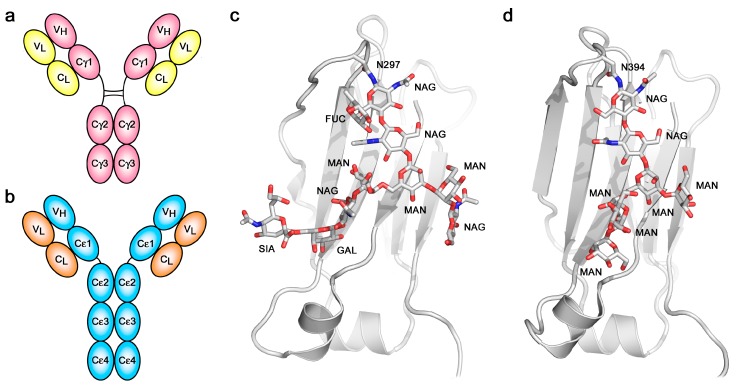
Overall structure and glycosylation. (**a**) Schematic representation of Immunoglobulin G (IgG). (**b**) Schematic representation of Immunoglobulin E (IgE). (**c**) The IgG Cγ2 domain contains complex carbohydrate covalently attached to Asn297 [26]. (**d**) The IgE Cε3 domain contains high-mannose carbohydrate covalently attached to Asn394 [27]. In panels (**c**,**d**), carbohydrate residues are labelled as follows: FUC, fucose; GAL, galactose; MAN, mannose; NAG, N-acetylglucosamine; SIA, sialic acid.

The overall architecture of the IgE molecule differs most significantly from that of IgG in respect to the “additional” heavy chain constant domain (Figure 1a,b) and the absence of a hinge region in the ε-chain. The six domains comprising the IgE-Fc, a dimer of Cε2-Cε3-Cε4 domains, are evolutionarily more ancient than the four-domain IgG-Fc. IgE-Fc resembles the (Cμ2-Cμ3-Cμ4)_2_ Fc structure of IgM, the most primitive antibody class, and the (Cυ2-Cυ3-Cυ4)_2_ Fc domains of avian IgY, the ancestor of IgE and IgG [24]. The hinge region of IgG appears to have evolved to take the place of the (Cε2)_2_ domain pair, since the Cγ2 and Cγ3 domains of IgG-Fc are most closely homologous to the Cε3 and Cε4 domains of IgE-Fc. IgM molecules, as pentameric or hexameric structures, are known to undergo conformational changes upon contact with antigen that dramatically alters the disposition of the Fab arms relative to the Fc region, as observed by electron microscopy (EM) [25]. Unliganded, the IgM molecules appear planar and “star-shaped”, while bound to the surface of antigens they form “table-like” structures with the Fab arms bent down and away from the Fc region. These observations are pertinent to the discussion of the flexibility and conformational change in IgE that will follow.

**Figure 2:**
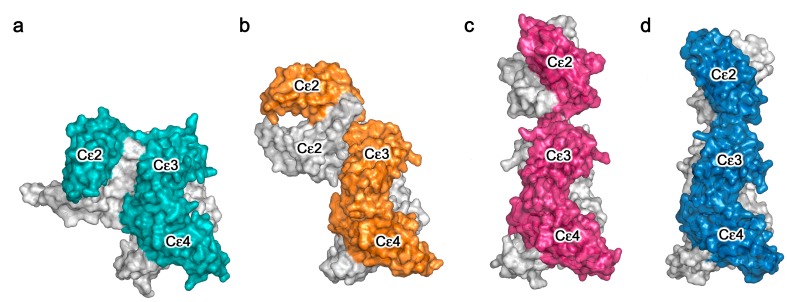
IgE-Fc is conformationally flexible. (**a**) Unbound IgE-Fc adopts an acutely bent conformation [34]. (**b**) IgE-Fc adopts a partially bent conformation when in complex with an omalizumab-derived Fab [36]. (**c**) Fully extended IgE-Fc conformation captured by aεFab [37]. (**d**) IgE-Fc adopts a fully extended conformation when in complex with the 8D6 Fab that is more compact than the conformation shown in (**c**) [38]. In panels (**a**–**d**), IgE-Fc chain B is coloured grey while chain A is coloured cyan, orange, pink and blue, respectively. For clarity, the anti-IgE Fabs are not shown in panels (**b**–**d**).

Expectations that IgE, with the additional domain pair, might adopt a more extended Y-shaped structure than that of IgG [28], were refuted by early biophysical studies of IgE in solution and when FcεRI-bound that indicated a more compact conformation [29,30]. In particular, elegant work with IgE molecules fluorescently labelled in their antigen-binding sites and at the C-termini of their Fc regions, clearly indicated through fluorescence (Förster) resonance energy transfer (FRET) distance measurements that the IgE molecule was not extended, but bent [31,32]. This was later confirmed by small-angle X-ray scattering (SAXS) studies of IgE and IgE-Fc in solution, the latter indicating that the Fc itself was a compact structure, best modelled by folding the (Cε2)_2_ domain pair back onto the Cε3-Cε4 domains [33]. However, when the first X-ray crystal structure of the whole IgE-Fc was solved [34], the bend was found to be even more acute than that which had been modelled (Figure 2a), with the Cε2 domain of one chain even contacting the Cε4 domain of the other; furthermore, by bending of the (Cε2)_2_ domain pair over towards one side of the (Cε3-Cε4)_2_ region, the IgE-Fc molecule adopted an asymmetrical three-dimensional structure, despite its symmetrical primary structure (chemical sequence). A FRET study of IgE-Fc further confirmed that this bent structure does indeed exist in solution [35]. Might IgE-Fc be able to “un-bend”, akin to the conformational changes that IgM appears to undergo?

Despite the identical primary structures of the two heavy (and two light) chains, IgE, like IgG and all other antibody classes, is glycosylated [39,40,41,42], and since there is heterogeneity not only in the pattern of glycosylation at the various potential sites but also in the composition at any particular site, the two heavy chains within any one IgE (or IgG) molecule are not precisely identical. Whether or not this compositional asymmetry is related to the asymmetric bending of the IgE-Fc has not been explored. One glycosylation site is conserved across all antibody classes: Asn394 in the Cε3 domain of IgE, structurally homologous to Asn297 in the Cγ2 domain of IgG. Other potential sites in the Cε2 and Cε3 domains are not always fully glycosylated, but Asn394, like its homologues in other antibody classes, is always fully occupied [39,40,41]. The branched carbohydrate chains occupy space between the Cε3 domains, as they do between the Cγ2 domains of IgG, but there is a major difference between IgE and IgG in this respect: the glycosylation at Asn394 in IgE is of the “high-mannose” type, in contrast to the “complex-type” at Asn297 in IgG (Figure 1c,d). Other glycosylation sites in IgE that are exposed at the surface are complex-type, which suggests that the high-mannose composition at Asn394 may be due to the Cε2 domains impeding access of the mannosidase enzymes responsible for trimming the high-mannose structures prior to assembly of the complex-type glycoforms. The same high-mannose structure is seen in IgY-Fc between the Cυ3 domains [43], perhaps similarly due to the presence of Cυ2 domains. The high-mannose, branched carbohydrate chains in IgE-Fc not only make non-covalent (hydrogen bond, hydrophobic and van der Waals) contacts with the Cε3 domains to which they are covalently attached, and to the adjacent Cε4 domains, but also make contact with each other, bridging the two heavy chains [27,34,44]. Despite this apparent structural role, and again in contrast to IgG in which loss of glycosylation at Asn297 compromises FcγR binding [45], both FcεRI and CD23 receptor-binding activity is maintained in the absence of glycosylation; IgE-Fc expressed in bacteria and refolded [46,47], or deglycosylated following mammalian expression [48,49], binds to both receptors. However, glycosylation at Asn394 is essential for the expression of functional IgE in mammalian cells in vitro and in vivo [41,50].

IgE thus differs in important ways from IgG, not only in terms of its overall structure and, as will now be discussed, its flexibility, but also with respect to the nature and the role of its glycosylation.

## 3. Conformational Dynamics in IgE-Fc

Crystal structures of the sub-fragment of IgE-Fc consisting of only the Cε3 and Cε4 domains, which we term Fcε3-4, and IgE-Fc, have revealed a degree of flexibility in the arrangement of the Cε3 domains relative to each other, either further apart (“open”) or closer together (“closed”) [27,34,36,37,38,44,51,52,53,54,55,56,57,58,59]. Furthermore, unliganded IgE-Fc structures were only bent (Figure 2a) [27,34,44]. It was therefore a considerable surprise to discover that in the crystal structure of the first complex between IgE-Fc and an anti-IgE antibody Fab, aεFab, the Fc had adopted a fully extended conformation (Figure 2c) [37]. Further analysis revealed that the anti-IgE Fab, which binds at the Cε2/Cε3 interface in a 2:1 complex with IgE-Fc, was selecting a pre-existing conformational state of the molecule in solution, and thus the question arose: if IgE-Fc could spontaneously “un-bend” to reach a fully extended state, could the (Cε2)_2_ domain pair then “flip over” to lie in a bent conformation on the other side of the Fcε3-4 region? In order to estimate the energetics of this potential “flipping” of the IgE-Fc, extensive molecular dynamics (MD) simulations were carried out [37]. It was discovered that the bent structure lies in a relatively deep energy well, but that once the IgE-Fc molecule had escaped this minimum, the “conformational landscape” was relatively flat, i.e., there were no significant barriers to prevent it reaching the extended conformation or indeed allowing the (Cε2)_2_ domains to bend over onto the other side of the molecule. The MD simulations revealed that this flipping of the Cε2 domains required the Cε3 domains to open somewhat, but the rate-limiting step for the process was clearly escape from the energy well representing the bent conformation. Most molecules would be in the bent state at any given time, consistent with the SAXS and FRET data in solution, but occasionally they flip over, although the rate and frequency of this event is difficult to assess.

**Figure 3:**
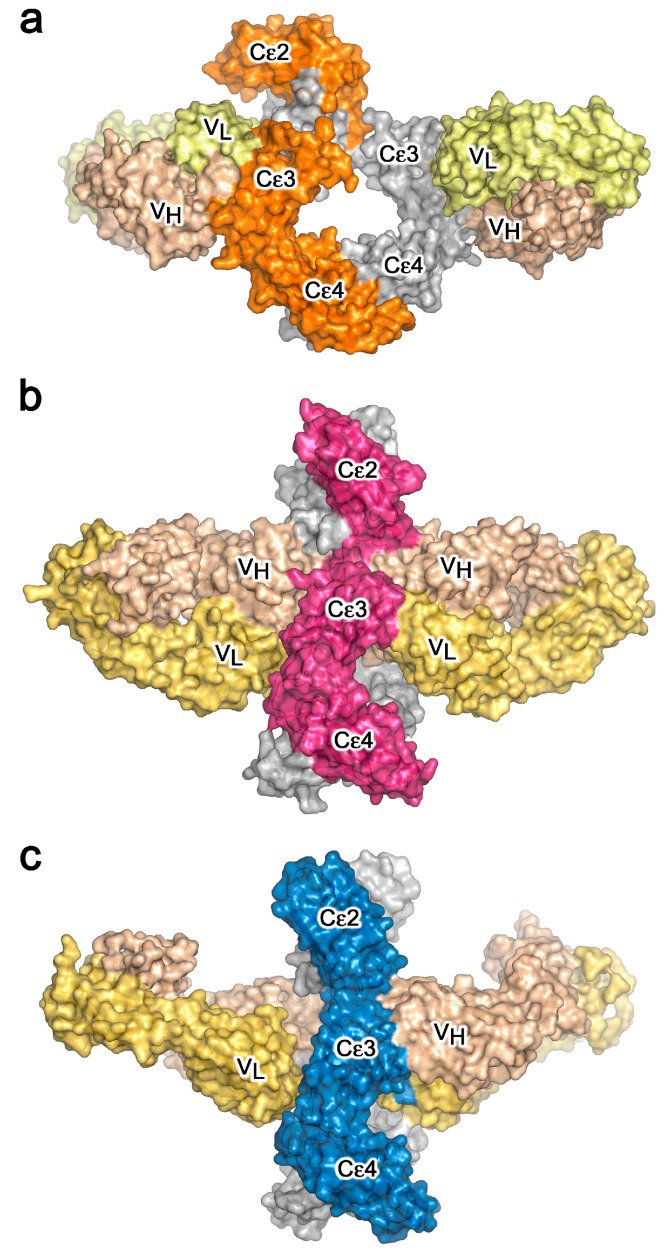
Crystal structures of IgE-Fc in complex with anti-IgE Fabs. (**a**) IgE-Fc in complex with an omalizumab-derived Fab [36]. (**b**) aεFab/IgE-Fc complex [37]. (**c**) 8D6 Fab/IgE-Fc complex [38]. In panels (**a**–**c**), IgE-Fc chain B is coloured grey while chain A is coloured orange, pink and blue, respectively. The Fab heavy and light chains are coloured in wheat and pale yellow, respectively.

Anti-IgE antibodies of the IgG class, such as aεFab, directed against the Fc region clearly have potential as anti-allergy therapeutics if, by either steric or allosteric means, they inhibit FcεRI or CD23 engagement. These activities will be discussed in the following two sections, and we first concentrate here on the lessons learned about IgE flexibility from structural studies of these anti-IgE Fab/IgE-Fc complexes. Omalizumab is a clinically approved anti-IgE antibody, and it binds to a partially bent conformation, intermediate between the bent and extended structures (Figure 2b) [36]. It binds to the Cε3 domains, also in a 2:1 complex, and causes the Cε3 domains to move further apart and adopt a very “open” conformation. Another anti-IgE antibody, termed 8D6, directed to the Cε2 and Cε3 domains, binds to a fully extended IgE-Fc conformation (rather like aεFab, Figure 2c) but in the 8D6 structure (Figure 2d) the (Cε2)_2_ domain pair is twisted and compressed towards the Cε3 domains, as in a corkscrew motion [38]. To date, these are the only structures that have been published for IgE-Fc in complex with anti-IgE Fabs (Figure 3).

The picture that emerges from these structural studies is that of a highly flexible Fc region in which the Cε2 domains are capable of extending and twisting relative to the Fcε3-4 region, or bending over to either side, with the Cε3 domains adopting closed or open states. With regard to the flexibility of the whole IgE molecule, i.e., that of the Fab arms relative to the Fc, we lack crystallographic data, although molecular simulations suggest that the short Cε1-Cε2 linker of only five or six amino-acids substantially restricts the available conformations compared with the Fab arm flexibility mediated by the hinge regions in IgG subclasses [35,37]. This is consistent with earlier biophysical studies in solution, which showed less Fab arm flexibility in IgE compared with IgG [60]. Nevertheless, despite lacking an IgG-like hinge, the linker between the Cε2 and Cε3 domains can clearly permit bending of the whole IgE molecule, just as is seen in IgM with its (Cμ2)_2_ domains and no hinge [25], although in IgM the precise nature of the bending remains unresolved.

## 4. IgE-Receptor Interactions

**Figure 4:**
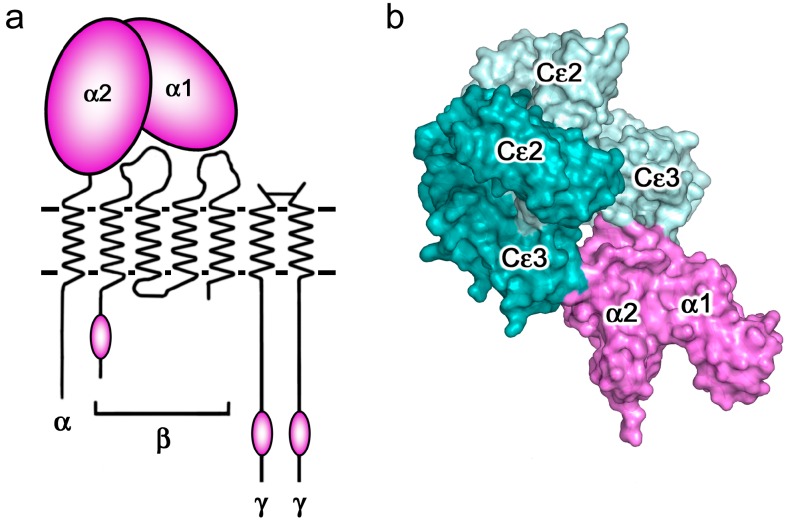
FcεRI (**a**) Schematic representation of FcεRI: the four chains are indicated, showing the two extracellular Ig-like domains of the α-chain that contain the IgE-binding activity, and the locations of the three intracellular ITAM signalling motifs. Figure adapted by permission from John Wiley & Sons, Inc. (Sutton, B.J.; Davies, A.M. Structure and dynamics of IgE-receptor interactions: FcεRI and CD23/FcεRII. *Immunol. Rev*. 2015, *268*, 222–235 [6]). (**b**) IgE-Fc adopts an acutely bent conformation when in complex with sFcεRIα, engaging the receptor (purple) at two distinct sub-sites [44]. IgE-Fc chains A and B are coloured dark cyan and pale cyan, respectively.

The structural details of IgE binding to the soluble extracellular domains of both FcεRI and CD23 are now well established. FcεRI expressed on mast cells and basophils comprises four polypeptide chains, αβγ_2_ (Figure 4a), but on other cell types it lacks the β-chain, which may serve either as an “amplifier” of down-stream signalling, since the β-chain contains an additional copy of the immuno-tyrosine activation motif (ITAM) present in the γ-chains, or it may affect surface expression [7]. All of the IgE-binding activity resides in the two Ig-like domains of the α-chain, termed sFcεRIα, the only substantial extracellular part of the receptor (Figure 4a). The crystal structure of sFcεRIα bound to Fcε3-4 first revealed the α2 domain and part of the α1-α2 linker bound across the two Cε3 domains, close to the point of connection to the Cε2 domains [56]. When the structure of the complex with the complete IgE-Fc was solved, contrary to expectations that the Fc might unbend, the angle was found to become even more acute (from 62° to 54°; Figure 4b) [44]. This enhanced bend seen in the crystal structure with IgE-Fc agrees not only with a recent study in solution with a FRET-labelled IgE-Fc molecule [35], but also, strikingly, with the work carried out more than 25 years ago with FRET-labelled IgE bound to FcεRI on cells, which showed a more compact structure for IgE when receptor-bound than in solution [32]. This orientation of IgE and acutely bent Fc, as indicated in Figure 4b, places constraints upon the disposition of the Fab arms, which may well be critical for understanding how the IgE molecule engages both FcεRI on cells and antigen (allergen), whether soluble or on a target cell, to enable receptor cross-linking and effector cell activation. These topological issues will be considered in more detail below.

**Figure 5:**
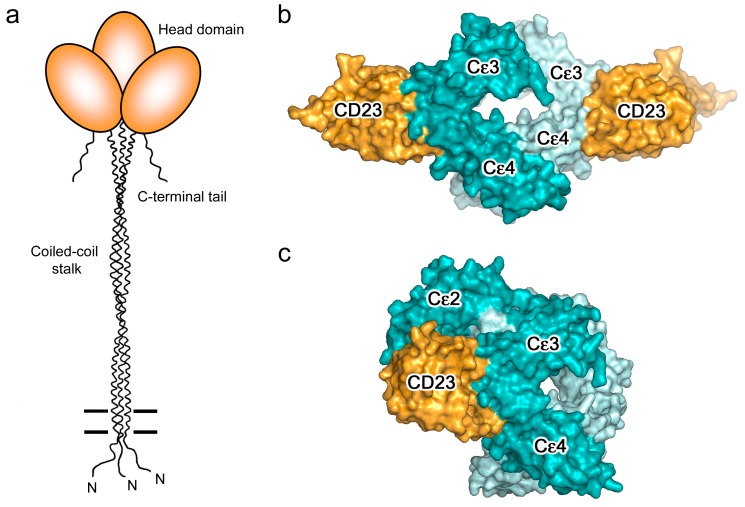
CD23. (**a**) Schematic representation of CD23: the three identical chains showing the triple α-helical coiled-coil “stalk”, C-type lectin-like IgE-binding “head” domains, and C-terminal “tails”. Figure adapted by permission from John Wiley & Sons, Inc. (Sutton, B.J.; Davies, A.M. Structure and dynamics of IgE-receptor interactions: FcεRI and CD23/FcεRII. *Immunol. Rev*. 2015, *268*, 222–235 [6]). (**b**) The 2:1 complex between sCD23 (orange) and Fcε3-4 [51]. (**c**) The 1:1 complex between sCD23 (orange) and IgE-Fc, in which IgE-Fc adopts an acutely bent conformation [53]. In panels (**b**,**c**), IgE-Fc chains A and B are coloured dark cyan and pale cyan, respectively.

CD23 is a homo-trimeric type-II membrane protein with its C-terminal C-type lectin-like “head” domains, to which IgE binds, spaced from the membrane by a triple α-helical coiled-coil “stalk” (Figure 5a). There is also a C-terminal “tail” of unknown structure that is required for binding to CD21, a co-receptor for CD23, the engagement of which is implicated in B cell activation and cell adhesion events [4,6,61,62,63]. We will focus on the IgE/CD23 interaction. The crystal structure of a single lectin-like domain alone, lacking the stalk and tail, which we will term sCD23, binds to IgE-Fc with a 2:1 stoichiometry, although the affinities for the two sCD23 molecules differ by more than a factor of ten (K_a_ ≈ 10^6^ M^−1^ and 10^5^ M^−1^) [53]. The binding of both molecules can be seen clearly in Figure 5b, one sCD23 molecule bound to each ε-chain in a similar manner, principally to Cε3 but also contacting Cε4, in this complex with Fcε3-4 [51]. However, the structure of sCD23 bound to IgE-Fc, which unexpectedly trapped only the first binding event (Figure 5c), explains the difference in affinity [53]. This 1:1 complex reveals how the first sCD23 molecule binds to an asymmetrically bent IgE-Fc, principally to Cε3 as before and also to Cε4, but with a single hydrogen bond and some van der Waals contacts with a Cε2 domain; the (Cε2)_2_ domain pair remains essentially bent, but swings about 16° to accommodate CD23 binding (Figure 5c) [53]. The site for the second CD23 head is completely accessible, although not occupied in this crystal structure, but this asymmetry of the two ε-chains explains the difference in affinity at the two CD23 binding sites.

As expected for a “C-type” lectin domain there is a Ca^2+^ binding site, although IgE binding does not require occupancy of this site [51,53,64]. Neither does this “lectin” interaction with IgE involve carbohydrate, although its binding to CD21 may be carbohydrate-dependent. In the presence of Ca^2+^, IgE binding is enhanced [62], 30-fold at 37 °C, through ordering of a loop and a subtle conformational change that enables additional contacts with IgE [54]. Intriguingly, these additional contact residues comprise a second Ca^2+^ binding site in murine CD23, an indication perhaps of a step in the evolution of the interaction of IgE with this C-type lectin domain. The Ca^2+^ dependence of the affinity, undoubtedly enhanced in the context of the trimer through an avidity effect, may be functionally important for unloading of IgE/allergen complexes by CD23 in endosomes, where the Ca^2+^ concentration is two to three orders of magnitude lower than at the cell surface, prior to CD23 recycling to the cell surface [65,66].

It is important to realise that although IgE can bind to two CD23 heads, these cannot belong to the same CD23 trimer; the N-termini of the two sCD23 molecules, which connect to the stalk (Figure 5b), are so far apart that most of the stalk would have to unravel for this to be possible [51]. However, IgE can cross-link two membrane CD23 trimers, and soluble trimeric forms of CD23 containing both head and stalk can cross-link membrane IgE (on B cells committed to IgE synthesis) or soluble IgE; in all of these cases, the bivalence of IgE and trivalence of CD23 can combine to create large complexes, which may be required for signalling in the context of B cell or APC activation [4].

## 5. IgE—An Allosteric Antibody

**Figure 6:**
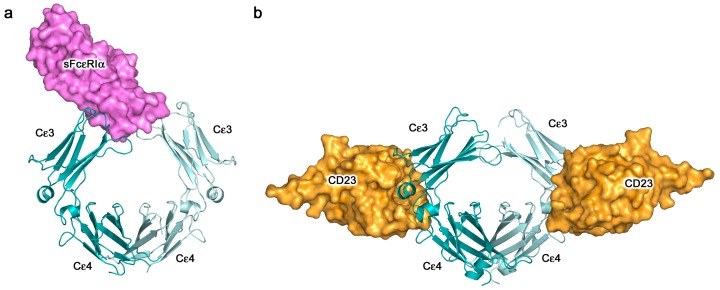
Binding of IgE to its receptors is allosterically regulated. (**a**) sFcεRIα (purple) binds to the Fcε3-4 region when the Cε3 domains adopt an open conformation [44]. (**b**) sCD23 (orange) binds to the Fcε3-4 region when the Cε3 domains adopt a closed conformation [51]. In panels (**a**,**b**), IgE-Fc chains A and B are coloured dark cyan and pale cyan, respectively.

The crystal structures of the two-receptor complexes reveal a key element of the IgE molecule, namely that there is allosteric communication between the two receptor-binding sites. It is known that IgE cannot bind to both receptors simultaneously [67,68], and vital that this is so, since otherwise trimeric CD23 could cross-link FcεRI-bound IgE on mast cells or basophils, causing activation and an inflammatory response in the absence of allergen. Indeed, binding of sFcεRIα inhibits sCD23 binding, and *vice versa* [51,69]. Earlier, it was thought that the two binding sites must overlap, but we know now that although both lie principally within Cε3, they are far apart from each other at opposite ends of the domain (Figure 4, Figure 5 and Figure 6). This mutual inhibition is achieved allosterically [51,69], mainly through changes in the disposition of the Cε3 domains relative to the Cε4 domains. To engage FcεRI, the Cε3 domains must adopt an “open” state (Figure 6a), which changes the angle between the Cε3 and Cε4 domains and prevents binding of CD23 at the Cε3/Cε4 interface. However, when CD23 binds, the Cε3 domains move closer together and this more “closed” conformation precludes FcεRI binding (Figure 6b).

Not only do the Cε3 domains undergo these domain motions, but they also appear to have evolved a high degree of intrinsic flexibility; when compared with other immunoglobulin domains in terms of hydrophobic core volume or other indicators of dynamics, Cε3 is clearly an outlier, and when expressed as an isolated domain it has been described as adopting a “molten globule” rather than a fully folded state [27,70,71,72,73,74]. Plasticity at the IgE-Fc/CD23 interface [55,75] and ordering of Cε3 upon FcεRIα binding [70] has been observed, with entropic contributions to the thermodynamics and kinetics of receptor binding playing an important role [44]. Remarkably, one of the earliest biophysical studies of IgE, not long after its discovery, identified the Cε3 domains as the most sensitive region of the molecule to heat denaturation [76], and this lability of Cε3 may in fact be critical for IgE’s unique receptor-binding properties and inter-site allosteric communication.

Allosteric effects in IgE-Fc were also observed when the mode of action of the anti-IgE omalizumab was elucidated through determination of the structure of the complex, and studies in solution [36]. It was discovered that omalizumab binding to IgE-Fc not only “unbends” the molecule as described above (Figure 2b), but causes the Cε3 domains to move so far apart that they cannot engage FcεRI, thus allosterically inhibiting FcεRI binding while simultaneously inhibiting CD23 binding orthosterically. Allostery and the conformational dynamics of IgE-Fc lie at the heart of a potentially even more important phenomenon concerning the inhibition of FcεRI binding; namely, the observation that it is possible for omalizumab not only to bind to free IgE and block binding to the receptor, but also to bind to receptor-bound IgE and facilitate its dissociation [36,77,78]. First reported with another IgE-Fc binding protein, a Designed Ankyrin Repeat Protein or Darpin [79], the ability of omalizumab to bind to FcεRI-bound IgE and cause it to dissociate was a most unexpected result, but one with exciting clinical potential. Although this “accelerated dissociation” only occurs at a very high concentration, above therapeutic levels of omalizumab [36,77], the explanation for this phenomenon lies in the fact that even when bound to FcεRI, IgE-Fc displays an ensemble of conformations; binding omalizumab alters the composition of this ensemble, reducing the energy barrier to IgE/FcεRI dissociation [36]. The intrinsic flexibility and allosteric properties of IgE can thus be exploited therapeutically to actively remove IgE from FcεRI.

Two other anti-IgE antibodies have been found to exploit allosteric effects. MEDI4212 inhibits FcεRI binding orthosterically and CD23 binding allosterically, the latter by locking the Cε3 domains in an open conformation [52]. Antibody 8D6, which extends the IgE-Fc as described above (Figure 2d), inhibits FcεRI binding both orthosterically and allosterically, but does not affect the CD23 interaction [38]; this may prove valuable therapeutically for allergic disease if down-regulation of IgE production can be effected through the interaction of 8D6/IgE complexes with mCD23 on B cells. The 8D6 antibody demonstrates that selective inhibition of IgE binding to its two principal receptors is possible.

## 6. Antigen (Allergen) Binding

So far, we have focussed on the Fc region of IgE and its receptor interactions. The binding of IgE to antigens, and in particular to allergenic proteins, has been studied in detail with antibody Fab fragments, but the flexibility of the IgE molecule as a whole, and in particular its ability to engage both allergen and its receptors, can only currently be inferred from low resolution electron microscopy (EM) studies and modelling; there are no high resolution structural data for intact IgE. EM studies of IgE complex formation with anti-idiotype IgG molecules have shown a relatively restricted degree of Fab arm flexibility [80], and a recent EM analysis of immune complex formation with IgE molecules binding to IgE epitopes grafted onto a small protein (myoglobin) framework, showed that the relative disposition, and in particular the proximity of the epitopes, affected immune complex formation and their ability to activate effector cells [81]. Modelling of Fab arm flexibility within the FcεRI-bound IgE molecule, confirmed this view that the relatively restricted range of dispositions of the Fabs, together with the particular geometrical arrangement of the epitopes on the allergen, might be key to an allergen’s potency in effector cell activation [35,37]. Other important requirements for a potent cellular response, in addition to epitope specificity, are affinity and the particular combination of antibodies present [82].

There are now several crystal structures of antibody Fabs binding to their specific epitopes on protein allergens, although most are murine IgG antibodies raised against the allergen [83,84,85,86,87,88,89,90]; not all of these may represent epitopes recognised by allergic patients’ IgE antibodies. Two studies generated IgE Fabs by phage display using combinatorial libraries derived from patients allergic to either the milk protein β-lactoglobulin (*Bos d* 5) [91] or the grass pollen allergen *Phl p* 2 [92], although these almost certainly do not consist of the “natural” V_H_-V_L_ pairing that occurred in the patient. A recent study generated a naturally paired V_H_-V_L_ combination by single B cell cloning of an IgG4 antibody from an allergic patient undergoing immunotherapy with the grass pollen allergen *Phl p* 7; this antibody was converted to an IgG1 Fab for the crystal structure analysis of the complex with allergen, and to IgE for functional analyses [23]. In all of these studies, the allergens were recognised by the antibodies in a conventional manner, involving many if not all of the CDRs. However, the most recent study also revealed an additional, unconventional “superantigen-like” interaction between *Phl p* 7 and the antibody, involving amino-acid residues of the V_L_ framework region (FR) [23].

**Figure 7:**
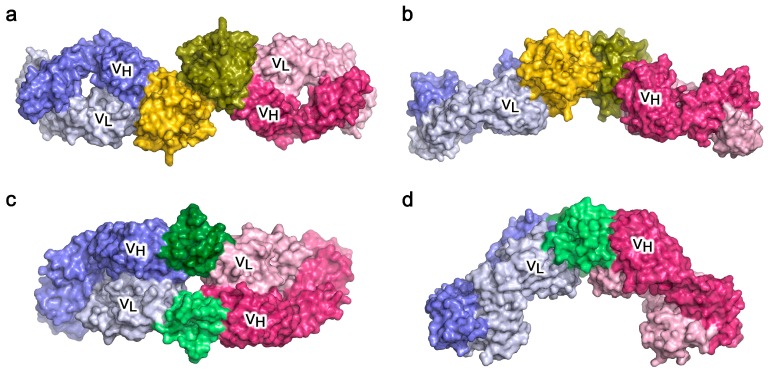
Crystal structures of allergens cross-linking two identical antibody Fab arms. (**a**) Dimer of allergen *Bos d* 5 (monomeric subunits coloured yellow and olive green) recognised classically by two identical Fab molecules (V_H_ and V_L_ domains indicated) [91]. (**b**) As a), orthogonal orientation [91]. (**c**) Two monomeric molecules of allergen *Phl p* 7 (coloured green), each independently recognised by two identical Fab molecules (V_H_ and V_L_ domains indicated) [23]. (**d**) As c), orthogonal orientation, in which only one of the two *Phl p* 7 molecules can be seen, recognised classically by the Fab on the right, and in a superantigen-like manner by the Fab on the left [23].

The allergen/antibody structures involving conventionally recognised epitopes demonstrate how an allergen that can dimerise, such as *Bos d* 5 [91], could cross-link two identical IgE antibodies (Figure 7a,b) and, if FcεRI-bound, lead to mast cell or basophil activation. A similar structure was seen in the complex of two identical Fabs bound to a dimer of the cockroach allergen *Bla g* 2 [86]; this allergen in monomeric form can however cross-link two antibodies that recognise epitopes on opposite faces of the allergen [93], and a similar topology arises for two different antibody Fabs that bind non-overlapping epitopes on the monomeric house dust mite allergen *Der p* 1 [89]. The non-conventional, partly FR-mediated recognition of *Phl p* 7 by an allergic patient’s antibody, occurring at the same time as conventional CDR-mediated recognition (Figure 7c,d), shows that certain allergens can cross-link identical IgE molecules using this alternative mechanism [23]. B cell superantigens, such as *Staphylococcus aureas* Protein A or *Peptostreptococcus magnus* Protein L, cross-link antibodies by interacting with their FRs, and thus molecules that cross-link IgE in this way, such as Protein L, have been termed “superallergens” [94]. *Phl p* 7 thus displays “superallergen-like” behaviour, which may contribute to the potency of particular allergens. Intriguingly, a structure of the monomeric cat allergen *Fel d* 1 in complex with an IgG Fab that blocks human IgE binding [90] shows a FR-mediated contact in the crystal which, together with the CDR-mediated interaction, could cross-link two identical Fabs in a manner very similar to that depicted for *Phl p* 7.

**Figure 8:**
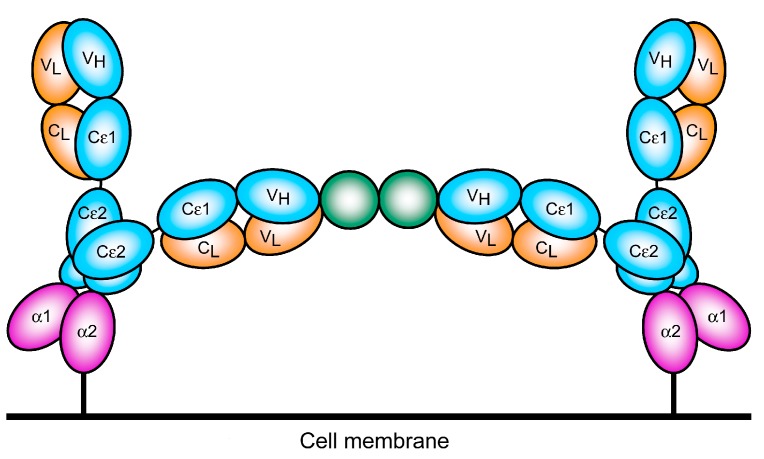
Schematic representation of FcεRI-bound IgE cross-linking by soluble allergen. A dimeric allergen (green) engages two identical IgE antibodies (blue and orange domains) that are bound by the Cε3 domains (Cε4 domains not shown) to the extracellular α1 and α2 domains of FcεRI (purple). This is representative of the structure shown in Figure 7a,b; a monomeric allergen could similarly cross-link two identical IgE molecules as shown in Figure 7c,d, or two different antibodies recognising non-overlapping epitopes. The restricted flexibility of the Fab arms in receptor-bound IgE may mean that the other arm is important for engagement of cell surface antigens.

Activation of mast cells or basophils by cross-linking FcεRI-bound IgE may thus be envisaged as shown in Figure 8. The regions of space accessible to the two Fab arms appear to be more restricted and almost non-overlapping when IgE is bound to the receptor: one arm points “parallel” to the membrane while the other points away [35,37]. These topological constraints may need to be considered when IgE is used to target cell surface antigens, rather than soluble allergens, to allow simultaneous engagement with FcεRI on effector cells.

## 7. Rationale for Harnessing IgE-Mediated Functions against Cancer

IgE is clearly a powerful activator of the immune system by virtue of the Fc receptor interactions described above, potentiating effector functions and antigen presentation; even well below receptor saturation levels, tissue-resident immune cells such as mast cells and macrophages enable this antibody isotype to exert long-lived and powerful immune surveillance in tissues such as the gut, skin, epithelial and mucosal surfaces. In addition to its contributions to the pathogenesis of allergic diseases and anaphylactic reactions, IgE plays a physiological role in immune protection against parasites, triggering inflammatory cascades that cause vasodilation and local enhancement of protective responses in conjunction with antibodies of other isotypes [95,96,97]. These latter, less well-described attributes of IgE may be of potential significance to applications in cancer immunotherapy.

### 7.1. Epidemiological Links between IgE, Allergy and Cancer

The concept of a role for IgE in conferring immune protection against cancer dates back many decades, with early studies providing evidence for a role of allergic responses in restricting tumour xenograft growth in mice, negative correlations between atopy and cancer [98,99,100,101,102], and decreased prevalence of immediate hypersensitivity in patients with cancer [103]. Immunohistochemical (IHC) evaluations on head and neck cancer showed that IgE-expressing cells were more abundant in tumours compared with normal mucosa [104], and a pancreatic cancer patient-derived IgE antibody could potentiate anti-tumour effector functions [105]. Certain conditions and stimuli that cause epithelial damage and stress signals may lead to the induction of an adaptive immune response favouring B cell class switching to IgE, which can restrict cancer growth. Such protective functions have been reported following local exposure of skin to environmental DNA-damaging stress signals, which triggered adaptive immune responses and the production of IgE antibodies that conferred protection from epithelial carcinogenesis [106]. Subsequent findings of inverse associations between allergic or atopic status and protection from cancer varied significantly. Inverse associations of allergic or atopic disease with the risk of developing specific malignancies including glioma, pancreatic cancer, lymphatic/hematopoietic, gastrointestinal, skin and gynaecological origin tumours have been reported [107,108,109,110,111], although significant limitations of such studies include the reliance of self-reported symptoms of allergy and lack of specific measurable biomarkers. More recent studies examined eosinophil counts and skin prick test positivity, as well as titres of IgE and allergen-specific IgE, with some reporting a reduced risk of developing specific cancers, and a reduced risk of developing cancer overall [110,111,112,113]. Although taken together, epidemiological reports point to the complex relationships between allergies, IgE levels and carcinogenesis, tantalising evidence also supports a functional role for IgE as a passive or active component in anti-tumour responses.

### 7.2. Features of IgE that may Translate to Immune Protective Functions against Tumours

To date, therapeutic monoclonal antibodies designed for the treatment of cancers are typically engineered with Fc regions belonging to the IgG isotype. IgG1 is typically chosen when effector functions are required, while IgG4 is preferred when Fc-mediated attributes are not desired. However, until recently, antibodies of other isotypes such as IgE or IgA had never been tested in humans [114,115,116].

In our studies, we hypothesised that several unique attributes of IgE could form a powerful immunological profile, suitable for the immunotherapy of solid tumours such as ovarian carcinomas [117]. These include high affinity for cognate receptors on a different set of immune cells to those engaged by IgG, long tissue residency and immune surveillance, the ability to potentiate strong effector functions at relatively low levels of Fc engagement with effector cells, and a lack of inhibitory Fc receptors.

*High affinity for cognate receptors:* The affinity of IgE for FcεRI is typically 100- to 10,000-fold higher than those of the clinically used IgG subclasses for their Fcγ receptors. Additionally, the avidity of IgE for trimeric CD23 is comparable to that measured with IgG-FcγRI complexes. These properties mean that IgE can persist on immune cells in the absence of antigen complex formation. If IgE antibodies are directed against cancer antigens, these features could be highly beneficial in ensuring potent effector functions, long persistence and immune surveillance at tumour sites.

*Lack of inhibitory Fc receptors:* IgE antibodies have no known inhibitory Fc receptors to moderate effector functions. This contrasts with IgG, which is subject to control by the inhibitory receptor, FcγRIIb, known to be upregulated in the tumour microenvironment (TME) of different cancer types. Lack of an inhibitory FcεR may mean that IgE is not subjected to suppressive influences imposed on IgG by tumours.

*Long immune surveillance in tissues:* The half-lives of IgE and IgG antibodies vastly differ in the circulation and tissues: 1.5 days for IgE and 2–3 weeks for IgG in the serum, partly due to the lack of FcRn binding by IgE. The opposite is true in tissues such as the skin, where the half-life of IgE is approximately two weeks compared with 2–3 days for IgG [118,119]. Long tissue residency and immune surveillance in the presence of FcεR-expressing effector cells could have potential benefits if directed against cancers, including epithelial and skin tumours such as malignant melanomas, squamous cell and ovarian carcinomas.

*Presence of IgE immune effector cells in tumours:* The inflammatory milieu of the TME may include FcεR-expressing immune effector cells such as monocytes, macrophages, mast cells, dendritic cells (DCs) and eosinophils. Although pro-tumoural or tumour-tolerant subsets of these cells may lack the ability to mount an anti-tumour attack, it is possible that cells armed by tumour antigen-specific IgE tightly bound on FcεRs could overcome tolerant phenotypes.

**Figure 9:**
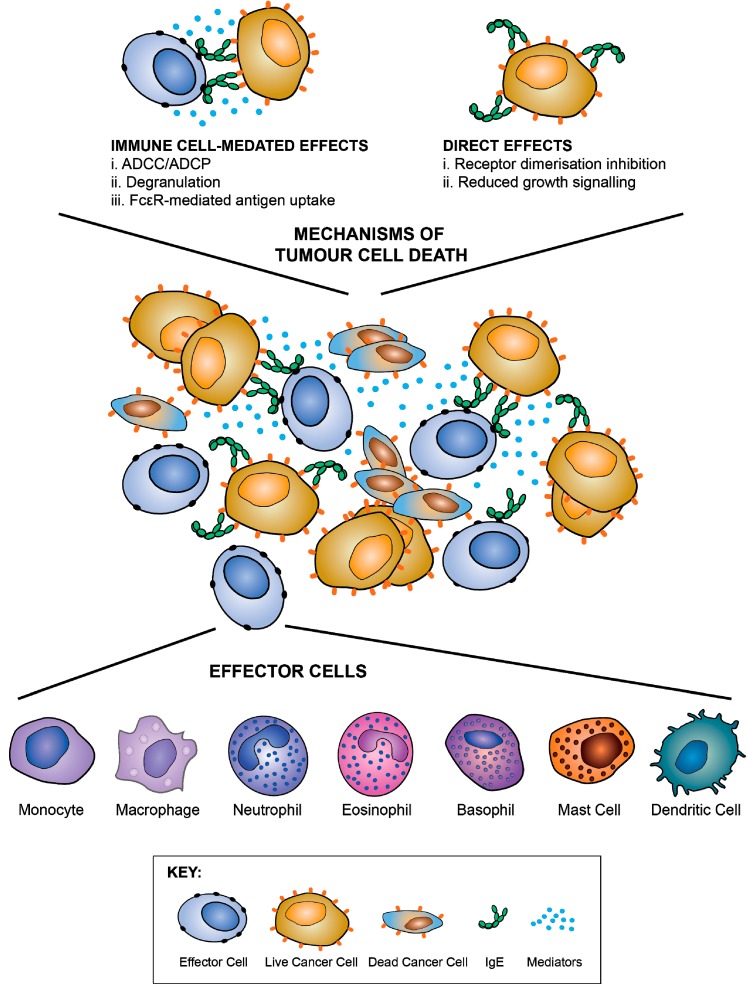
IgE functions against cancer cells. IgE can potentiate Fc-mediated effector functions by engaging cognate receptors on immune effector cells such as monocytes, macrophages, neutrophils, eosinophils, basophils and mast cells. Antibody-dependent cell-mediated cytotoxicity (ADCC), and degranulation can result in the release of various toxic and pro-inflammatory mediators, including proteases, cytokines, chemokines, and histamine, which, together with antibody-dependent cell-mediated phagocytosis (ADCP), can result in enhanced anti-tumour functions and immune cell recruitment. IgE can also engage APCs to enhance antigen uptake and presentation. Like anti-cancer IgG antibodies, IgE may also exhibit direct effects against cancer cells, such as receptor dimerisation inhibition and reductions in cancer cell growth signalling. Figure adapted by permission from Taylor & Francis (Josephs, D.H. et al. IgE immunotherapy: a novel concept with promise for the treatment of cancer. *mAbs* 2014, *6*, 54–72 [117]) and John Wiley & Sons, Inc. (Jensen-Jarolim, E. et al. AllergoOncology - the impact of allergy in oncology: EAACI position paper. *Allergy* 2017, *72*, 866–887 [137]).

*Fc-mediated effector functions:* IgE can potentiate a range of effector functions through the engagement of FcεRI and CD23. These include: antibody-dependent cell-mediated cytotoxicity (ADCC) by immune cell types including monocytes, macrophages, eosinophils and mast cells, with the release of toxic mediators (e.g., nitric oxide), proteases, cytokines and chemokines (e.g., tumour necrosis factor, TNFα, macrophage chemoattractant protein-1, MCP-1) associated with target cell lysis; antibody-dependent cell-mediated phagocytosis (ADCP) by macrophages and monocytes; mast cell and basophil degranulation leading to the release of proinflammatory mediators, and the enhancement of immune cell recruitment and activation at the antigen challenge sites (Figure 9). These attributes could result in enhanced immune cell recruitment, surveillance and anti-tumour functions.

*Exerting anti-parasite effector functions:* The physiological roles of IgE in protective immune responses against parasites are well documented. Anti-parasitic IgE and IgE loaded on effector cells such as eosinophils have been shown to confer protection against different parasites (e.g., *Schistosoma mansoni*) [120]. IgE engaged with FcεRI or CD23 can engender parasite clearance by human eosinophils, platelets and macrophages through ADCC and ADCP [121,122]. Furthermore, high serum titres of parasite antigen-specific IgE have been associated with resistance to infection [123,124]. Macrophages, eosinophils and mast cells have all been reported to be involved in these protective mechanisms [5,97,122,125,126]. IgE-mediated immune clearance of large parasites in tissues, including Th2-biased environments such as the gut, draw parallels with conditions in solid tumours in which a similar Th2 inflammatory milieu and the presence of immune cells such as macrophages may form appropriate environments in which IgE could act to eradicate tumours by similar mechanisms.

*Overcoming antibody blockade mechanisms associated with Th2-biased tumour conditions:* Tumour-associated production of alternatively activated (e.g., IL-10-driven) rather than classically activated (IL-4-driven) Th2 environments may support local antibody class switching to inflammatory and immunologically inert subclasses such as IgG4. Th2-biased inflammatory states that favour B cell class switching to IgG4 have long been identified in IgG4-related diseases characterised by chronic inflammation, circulating IgG-positive plasmablasts and high infiltration of IgG4-producing plasma cells in various tissues [127,128,129]. Alternative Th2 activation states have also been reported in several solid tumour types including pancreatic cancer, extrahepatic cholangiocarcinoma, melanoma and non-small cell lung cancer [130,131,132,133,134]. These pathological conditions, likely to be promoted by a combination of a Th2-biased inflammatory milieu and long antigen exposure, may signify that immune responses are driven away from the classical Th2-based class switching to IgE, in favour of IgG4. Evidence points to IgG4 antibodies not only being immunologically inert, but importantly, being able to impair the immune-activating functions of otherwise cytotoxic IgG1 antibodies [134,135]. Numerous mechanisms may be at play, including competition for FcγR engagement with other IgGs, Fab arm exchange, and signalling through inhibitory Fc receptors, all supporting immunosuppressive signals [130,136]. The latter could have implications not only for modulating the endogenous humoral immune response but also for restricting the potency of IgG1 therapies. These regulatory mechanisms may offer opportunities to design anti-tumour IgE antibodies that function through a different Fc receptor, which could be less prone to the immunosuppressive signals that impair IgG functions against cancer.

*Engaging antigen presenting cells to stimulate effective adaptive immune response:* IgE can engage with APCs to enhance antigen uptake and presentation to cognate T cells (Figure 9). IgE engagement with FcεRI can cross-present antigen, priming a cytotoxic T lymphocyte (CTL) response [138,139]. Through such mechanisms, IgE has been reported to confer protective anti-tumour immunity and trigger memory responses. These antigen presentation-boosting attributes could be important in the TME where the functions and maturation of professional antigen presenting cells may be impaired.

## 8. Pre-Clinical Studies of IgE Antibodies Targeting Cancer Antigens: The Advent of Allergo Oncology

The development of immunologically active, antibody-based targeted therapies with potent Fc-mediated effector mechanisms has revolutionized the treatment of cancer patients with previously difficult to treat tumours [140]. A promising branch of this discipline is the emerging field of AllergoOncology, which focuses on Th2 and IgE-mediated immune responses in the cancer context [137,141,142,143]. Research in this field has opened the way for the development of IgE-based immunotherapy approaches, including monoclonal IgE antibodies as anti-cancer treatments [117,144].

The specific attributes of IgE described above, including natural immune activatory functions in tissues and high affinity for cognate receptors, have been proposed as a strategy for cancer immunotherapy. Antibodies engineered with IgE Fc regions, and designed to recognise tumour-associated antigens, may promote immune cell recruitment into tumours, and both direct and activate the Th2-biased immune stroma against cancer. Longer tissue-resident immune surveillance may then translate to anti-cancer efficacy. Therapeutic approaches have been developed to harness the immune-activating functions of IgE for cancer immunotherapy, including: IgE-coated cell vaccines, IgEs as adjuvants, vaccination approaches to trigger IgE-biased immune responses against tumour antigens, and recombinant IgE recognising tumour antigens. Here, we will focus on the development of recombinant IgE antibodies [144]. Furthermore, we place specific emphasis on MOv18 IgE, as the first-in-class agent that has undergone extensive pre-clinical efficacy and safety evaluations in several model systems, prior to reaching clinical testing in patients with cancer.

### 8.1. Engineering Platforms for Production of IgE Antibodies for Research and Clinical Translation

Developing IgE antibodies that recognise cancer antigens relies on appropriate expression systems and protocols to facilitate antibody cloning and production. Since the development of hybridoma technology five decades ago, novel recombinant DNA technology, genetic manipulation and advances in cell biology have led to remarkable improvements in therapeutic recombinant antibody engineering [145]. Although significant efforts have focused on the optimization of expression platforms for IgG [146], relatively meagre investment has been directed towards engineering IgE.

The study and clinical translation of IgE antibodies requires efficient and scalable production processes, but these have historically been characterised by low and variable yields. Despite this, several groups have shown that recombinant IgE antibodies can be produced using various cloning strategies. In early studies, restriction enzyme-based cloning methodologies were successfully employed using murine expression host cells to derive stable expression platforms, with Sp2/0 [147] and FreeStyle^TM^-293F [148] cell lines, reaching production yields in the range of 8–25 mg/L. Recombinant IgE antibodies have also been produced using transient expression platforms with human (HEK293T, FreeStyle^TM^-293F, Expi293F^TM^ cells), insect- and plant-based systems, reaching yields of 30 mg/L [41,82,149,150]. More recent transient expression protocols have been implemented, which take advantage of Polymerase Incomplete Primer Extension (PIPE) cloning [151]. PIPE does not rely on restriction or other recombination sites, and can help expedite antibody cloning, a strategy that we have applied to IgE antibody production [152].

We recently developed a highly expressing stable recombinant IgE expression system for rapid production of a functional antibody, with features that allow scale-up for potential clinical evaluations [153]. For this, we implemented PIPE cloning and generated a vector containing the Ubiquitous Chromatin Opening Elements (UCOE) sequence located upstream of the transgene promoter to prevent promoter silencing. UCOE allows the expression of the transgene even if it is randomly integrated in a heterochromatin region [154]. This platform improves IgE yields to 87 mg/L per day, at least 33-fold higher production within four days compared with the best stable IgE expression system documented to date, and in small culture volumes of 25 mL, with the potential for further scale-up production.

These findings suggest that, as with IgG antibody production, IgE can be produced using a range of expression systems and with sufficient yields to facilitate the functional evaluation and translation to clinical testing. Further efforts in the field promise to improve upon existing platforms for use in pre-clinical studies, process development, Good Manufacturing Practice (GMP) production and supply of material suitable for clinical studies. Other developments in antibody discovery such as knock-in mouse strains used to derive IgE antibodies by hybridoma techniques, phage display approaches using human antibody variable region repertoire libraries and single B cell cloning techniques may also be applicable [155,156,157].

Recombinant IgE antibody production has advanced significantly with several already engineered and tested in vitro and in vivo. There is however room for further development of improved and effective production systems that can be translatable to GMP environments and scale-up for clinical studies.

### 8.2. Functional Evaluations of Anti-Tumour IgEs

#### 8.2.1. In Vitro and In Vivo Functional Profiles of Engineered IgEs Targeting Several Cancer Antigens

Antibody engineering has yielded the first generation of IgE antibodies that have been studied in vitro and in vivo in numerous model systems. Anti-tumour IgE antibodies can engage various immune effector cells such as mast cells and basophils expressing high levels of tetrameric FcεRI (αβγ_2_), and monocytes and eosinophils that express trimeric FcεRI (αγ_2_) at lower levels. Studies in vivo have been conducted in various mouse immunocompetent models. However, human IgE-Fc does not cross-react with mouse FcεR and, unlike in humans, mouse FcεRs are only expressed by mast cells and basophils, making the mouse immune system less suitable for the study of human IgE functions. However, transgenic mouse models have shown significant tumour-restricting abilities of IgE with human Fc domains. Examples of several monoclonal IgE antibodies evaluated over the last 30 years are discussed below.

A mouse IgE recognising the mammary tumour virus (MMTV) major envelope glycoprotein (gp36) was tested in an immunocompetent syngeneic mammary carcinoma. The antibody restricted the growth of subcutaneous (s.c.) mammary tumours compared with controls [158]. Another murine IgE recognising a colorectal cancer antigen (CCA) restricted the growth of an s.c. tumour in an antigen-specific and species-specific manner at concentrations far lower than those required for the equivalent IgG to engender the same effect [159]. A fully human anti-HER2/*neu* IgE (C6MH3-B1 IgE) restricted the growth of intraperitoneal (i.p.) tumours compared to vehicle controls and prolonged the survival of human FcεRIα-transgenic mice [160]. The same agent was well tolerated when administered in cynomolgus monkeys, albeit at very low doses (up to 80 µg/kg). Another IgE specific for the epithelial tumour antigen MUC-1 restricted cancer growth when expressed locally in tumours along with chemoattractant mediators MCP-1 and IL-5 [161]. Furthermore, a mouse/human chimeric IgE antibody (clone AR47.47) recognising the prostate specific antigen (PSA) enhanced antigen presentation by DCs, and triggered CD4+ and CD8+ T cell responses. The same antibody complexed with its antigen prolonged the survival of human FcεRIα-transgenic mice subsequently challenged with prostate cancer cells [162].

Human/mouse chimeric anti-HER2/*neu* IgE, and anti-EGFR (epidermal growth factor receptor) IgE, engineered from the original trastuzumab and cetuximab (IgG1) clones respectively, were shown to engender ADCC by human monocytic cells [163,164]. Specifically, anti-EGFR IgE triggered superior ADCC functions (70%) against cancer cells, compared with the corresponding IgG1 (30%) [164]. However, some episodes of anaphylaxis were observed in some patients with EGFR-positive tumours who received the anti-EGFR human/chimeric monoclonal IgG1 antibody cetuximab. These were caused by the presence of pre-existing IgE antibodies specific for the oligosaccharide galactose-α-1,3-galactose (α-Gal) on SP2/0-expressed cetuximab in a subset of individuals [165,166]. Furthermore, humans are known to carry IgG and IgM antibodies recognising α-Gal [167], and it is possible that these endogenous antibodies could have neutralised the anti-tumoural effects of cetuximab. Therefore, caution should be exercised in translating IgE class antibodies recognising EGFR on the grounds of safety and efficacy. An anti-human CD20 IgE triggered eosinophil-mediated ADCC and mast cell activation and killing of CD20-expressing tumour cells. Anti-HER2/*neu*, anti-EGFR, anti-CD20, anti-folate receptor alpha (FRα) IgE and anti-prostate specific antigen (PSA) IgE antibodies were all able to trigger rat basophil leukaemia (RBL) SX-38 mast cell degranulation when cross-linked in different ways including soluble antigen/polyclonal antibody complexes, cancer cells expressing multiple copies of the target antigen, and polyclonal anti-IgE. Furthermore, anti-HER2/*neu* (trastuzumab) IgE demonstrated the ability to exert direct effects on tumour cell viability in the absence of effector cells, equivalent to those reported to be triggered by trastuzumab IgG [163]. This supports the notion that anti-tumour IgE antibodies may be capable of engendering direct effects attributed to IgG equivalent agents, whilst perhaps still able to harness class-specific effector functions (Figure 9).

The progress of the first-in-class monoclonal IgE antibody (MOv18) recognising a tumour-associated antigen to an early clinical trial in oncology is the exemplar advance in the field. Based on this development, herein we will focus on the evaluation and translation of this recombinant antibody, and efforts to translate IgE class therapeutic agents to clinical testing. If firstly safety, and secondly efficacy of this first-in-class agent could be demonstrated in the clinic, this will pave the way for further study and translation of the above-mentioned antibodies, as well as other novel anti-cancer antibodies of this class.

#### 8.2.2. MOv18 IgE, the First Anti-Tumour IgE to Reach Clinical Testing: Evaluation of In Vitro Effector Functions

An IgE antibody that has progressed to clinical testing is MOv18, a mouse/human chimeric monoclonal IgE antibody that recognises the tumour-associated antigen Folate Receptor alpha (FRα) (NCT02546921, www.clinicaltrials.gov). FRα is highly expressed in > 70% of ovarian carcinomas and other tumour types and has low and restricted expression distribution in normal tissues [168,169]. The IgG1 version of MOv18 has undergone early clinical trials as a therapeutic and imaging agent in patients with ovarian carcinomas, and treatment has been well tolerated [170,171,172,173]. FRα is considered a promising target for cancer therapy, with considerable evidence that either directing therapeutic antibodies to this receptor, or its inhibition by small molecules, is well-tolerated in man [174,175,176,177,178].

**Figure 10:**
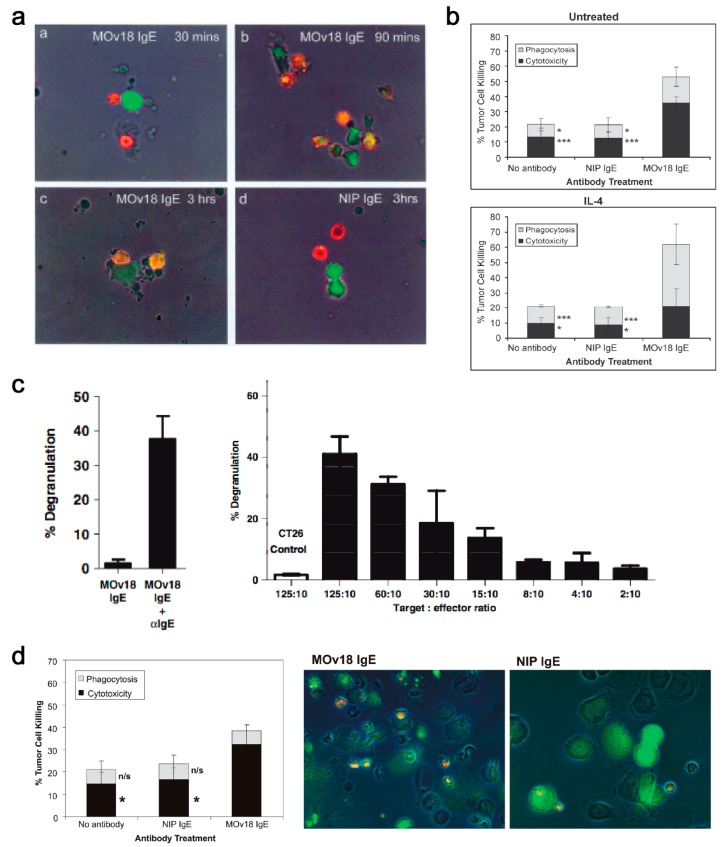
In vitro evaluations of MOv18 IgE. (**a**) Live imaging studies showed contact between IGROV1 ovarian cancer cells and CD14-labelled human monocytes within 30 min of incubation of PBMCs and IGROV1 cells together with MOv18 IgE. Following 90 min, the phagocytosis of tumour cells was evident and IGROV1 cells became fragmented by 3 h [179]. Figure adapted by permission from John Wiley & Sons, Inc. (Karagiannis, S.N. et al. Activity of human monocytes in IgE antibody-dependent surveillance and killing of ovarian tumor cells. *Eur. J. Immunol*. 2003, *33*, 1030–1040 [179]). (**b**) Human monocytes expressing cell-surface FcεRI triggered MOv18 IgE-mediated ADCC of IGROV1 ovarian cancer cells, and IL-4 stimulated monocytes with up-regulated CD23 expression, killed tumour cells by both ADCC and ADCP compared to background levels mediated by non-specific NIP IgE and no IgE controls [180]. Figure adapted by permission from Springer Nature. (Karagiannis, S.N. et al. Role of IgE receptors in IgE antibody-dependent cytotoxicity and phagocytosis of ovarian tumor cells by human monocytic cells. *Cancer Immunol. Immunother.* 2008, *57*, 247–263 [180]). (**c**) Appreciable degranulation of RBL SX-38 cells was triggered by cross-linking of cell surface receptor-bound MOv18 IgE by polyclonal anti-IgE antibody (left) or FRα-expressing cancer cells (right) [182]. Figure adapted by permission from John Wiley & Sons, Inc. (Rudman, S.M. et al. Harnessing engineered antibodies of the IgE class to combat malignancy: initial assessment of FcɛRI-mediated basophil activation by a tumour-specific IgE antibody to evaluate the risk of type I hypersensitivity. *Clin. Exp. Allergy*, 2011, *41*, 1400–1413 [182]). (**d**) MOv18 IgE-mediated killing of IGROV1 ovarian cancer cells by primary human eosinophils (right) and microscopic evaluations revealed interactions between IGROV1 cells and eosinophils, and IGROV1 tumour cell destruction alongside piecemeal degranulation of eosinophils, following 2.5 h of incubation with MOv18 IgE, but not with non-specific NIP IgE (right) [181]. Figure adapted by permission from The American Association of Immunologists, Inc. (Karagiannis, S.N. et al. IgE-antibody-dependent immunotherapy of solid tumors: cytotoxic and phagocytic mechanisms of eradication of ovarian cancer cells. *J. Immunol*. 2007, *179*, 2832–2843 [181]).

In vitro, mouse/human chimeric MOv18 IgE activated human peripheral blood mononuclear cells (PBMCs) to kill ovarian cancer cells, compared with background cancer cell death with nonspecific mouse/human chimeric anti-4-hydroxy-3-nitro-phenacetyl (NIP) IgE, or no antibody controls [179]. Human monocytes were subsequently identified as important effector cells in PBMCs, based on live imaging studies in which IGROV1 ovarian cancer cells were found to contact one or more CD14-labelled human monocytes within 30 min of incubation of PBMCs and IGROV1 cells together with MOv18 IgE. Phagocytosis of tumour cells was evident after 90 min of incubation, with IGROV1 cells becoming fragmented by 3 h (Figure 10a).

Following stimulation by IL-4, which is often released from IgE-sensitized basophils and mast cells, CD23 can be upregulated on monocytes, eosinophils and platelets. Interaction of IgE with CD23 may also have a role in ADCP of target cells by effector cells, as shown by its natural protective role in the clearance of parasites. This function has also been described with MOv18 IgE. Human monocytes expressing FcεRI on the cell surface triggered IgE-mediated ADCC of tumour cells, while IL-4 stimulated monocytes killed FRα-expressing tumour cells by both ADCC and ADCP, compared to background levels of tumour cell death with NIP IgE and no IgE controls (Figure 10b). Specific IgE Fc receptor blockade studies in vitro confirmed that MOv18 IgE-dependent ovarian tumour cell killing had an ADCC component, primarily mediated by FcεRl, and an ADCP component, primarily mediated by CD23 [180,181].

The ability of MOv18 IgE to trigger functional degranulation was examined with RBL SX-38 cells engineered to over-express the human tetrameric FcεRI. Exposure of the RBL SX-38 cells to MOv18 IgE alone did not induce significant degranulation; however cross-linking MOv18 IgE bound to the effector cell surface using either a polyclonal anti-IgE antibody or FRα-expressing cancer cells induced appreciable degranulation (Figure 10c) [182]. Eosinophils are key IgE effector cell types known to express low levels of FcεRI, but not CD23 [183]. Eosinophils mediated elevated ADCC (32.4%) with MOv18 IgE above isotype controls, and microscopical evaluations revealed contact between eosinophils and tumour cells, frequently accompanied by eosinophil degranulation, loss of tumour cell architecture, and apparent tumour cell death (Figure 10d) [181]. Our findings were consistent with data by Teo and colleagues who also reported the eosinophil-mediated ADCC functions by an anti-CD20 IgE antibody [161]. Interestingly, previous studies showed a lack of eosinophil activation by IgE cross-linked with allergens. These differences could relate to the density of the target antigen. Tumour cells express very high numbers of tumour associated-antigens on their surface, crosslinking of which may be required to deliver an activatory signal through the lowly expressed FcεRI on eosinophils. However, this may not be the case for the crosslinking of FcεRI by IgE complexed with multivalent allergens of a much lower valency [184]. In the cancer context, the target antigen density could therefore be critical to triggering eosinophil-mediated anti-tumour IgE effector functions.

These studies established that MOv18 IgE could mediate effector functions such as degranulation and tumour cell killing through cytotoxicity (ADCC) and phagocytosis (ADCP) by activating known IgE effector cells.

#### 8.2.3. In vivo efficacy studies of MOv18 IgE

The ability of MOv18 IgE to restrict tumour growth in vivo was studied against different rodent models including human tumour xenografts established in immunodeficient (SCID and nu/nu) mice. In immunodeficient mouse models, human effector cell populations were co-administered with MOv18 IgE because: (a) human IgE-Fc is not recognised by mouse FcεRs, and (b) in mice the high-affinity IgE receptor FcεRI is expressed only by mast cells and basophils, and is absent in key effector cells such as monocytes and eosinophils. These studies therefore took place in an in vivo system containing both target and effector cells of human origin.

**Figure 11:**
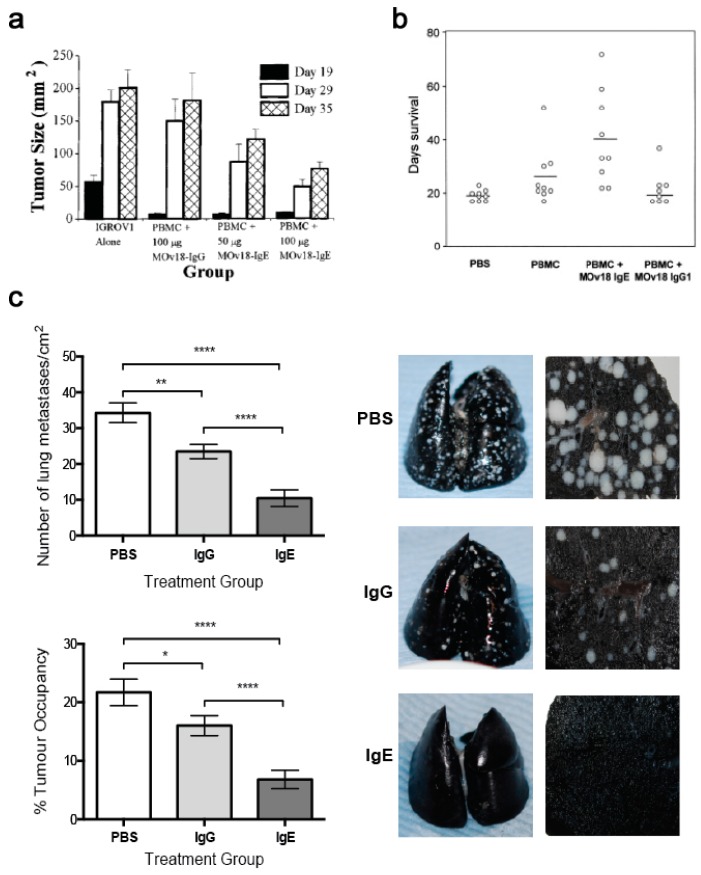
In vivo evaluations of MOv18 IgE. (**a**) In an s.c. human ovarian cancer (IGROV1) xenograft grown in a SCID mouse model, reduced tumour growth was measured in animals treated with PBMC plus MOv18 IgE, even at day 35 post tumour challenge. In comparison, animals treated with PBMC plus MOv18 IgG1 showed initial inhibition of tumour growth at day 19, but by day 35 tumours grew to the same size as the controls [147]. Figure adapted by permission from John Wiley & Sons, Inc. (Gould, H.J. et al. Comparison of IgE and IgG antibody-dependent cytotoxicity in vitro and in a SCID mouse xenograft model of ovarian carcinoma. *Eur. J. Immunol*. 1999, *29*, 3527–3537 [147]). (**b**) In an orthotopically-grown (i.p.) patient-derived xenograft (PDX) model of ovarian cancer, mice treated with weekly doses of PBMC plus MOv18 IgE showed superior survival compared to untreated animals and those treated with PBMC alone or PBMC plus MOv18 IgG [179]. Figure adapted by permission from John Wiley & Sons, Inc. (Karagiannis, S.N. et al. Activity of human monocytes in IgE antibody-dependent surveillance and the killing of ovarian tumor cells. *Eur. J. Immunol*. 2003, *33*, 1030–1040 [179]). (**c**) Left panel: In an immunocompetent syngeneic tumour model in WAG rats, significantly superior tumour growth restriction was measured in animals treated fortnightly with 10 mg/kg rat MOv18 IgE compared to the rat IgG2b equivalent. Right panel: Representative images of Indian ink-stained rat lungs (left) and lung sections (right) from each treatment group are shown [186]. Figure adapted by permission from the American Association for Cancer Research. (Josephs, D.H. et al. Anti-Folate Receptor-α IgE but not IgG Recruits Macrophages to Attack Tumors via TNFα/MCP-1 Signaling. *Cancer Res*. 2017, *77*, 1127–1141 [186]).

In an s.c. human ovarian cancer (IGROV1) xenograft grown in a SCID mouse model, animals administered with mouse/human chimeric MOv18 IgE or MOv18 IgG1, intravenously (i.v.) exhibited an initial inhibition of tumour growth up to day 19 post-tumour challenge. However, the tumours in mice administered PBMCs and MOv18 IgG1 subsequently grew to the same size as the controls. In contrast, mice administered PBMCs and MOv18 IgE exhibited reduced growth of up to 72% by day 35 post-challenge. In a range of experiments in this model, a single treatment with MOv18 IgE and PBMC significantly restricted the growth of ovarian tumours (Figure 11a) [147]. In specimens sampled at the end of these studies, tumours from the mice that received PMBCs and MOv18 IgE showed significantly larger areas of necrosis compared with those from mice treated with non-specific control IgE plus PBMCs, or those given PBMCs alone. Furthermore, when administered to IGROV1 xenograft mice in the absence of human PBMC, MOv18 IgE did not significantly inhibit tumour growth. Therefore, in the IGROV1 xenograft model, the anti-tumour efficacy of MOv18 IgE was found to be reliant on the presence of both an effector cell population and an IgE targeted to a tumour-expressed antigen.

Subsequently, a patient-derived xenograft (PDX) model of ovarian cancer was established from a human primary tumour sample, originating from the ascites of a moderately differentiated Grade 3, stage III ovarian serous cystadenocarcinoma. This PDX could be passaged in nude mice while retaining its human phenotype and was found to express FRα. In efficacy studies using this model, nude mice were challenged with i.p. ascites from donor human xenograft-bearing mice and were then treated with saline, human PBMCs or PBMCs plus MOv18 IgE on days 1 and 16. The mean survival time of control mice was 22 days, for those administered PBMCs alone it was 30 days, while for those administered PBMCs plus MOv18 IgE, the mean survival time was 40 days [179]. In a study comparing the efficacy of weekly doses of MOv18 IgG and IgE in this model, untreated mice survived for a median of 19 days, those administered PBMCs alone survived for 26 days, those administered PBMC plus IgG1 survived for 22 days, and those administered PBMC plus IgE survived for 40 days (Figure 11b).

One limitation of studies in mouse models is the need to introduce exogenous human effector cells, thus limiting the immune functions of the model and the possible duration of study as exogenous effector cells become depleted. Therefore, an immunocompetent syngeneic tumour model in Wistar Albino Glaxo (WAG) rats was designed to study efficacy as well as safety of MOv18 IgE prior to clinical translation. This model was selected based on similar expression and cellular distribution of FcεRI in rats and humans. Rat CC531 colon adenocarcinoma cells [185], engineered to express the human FRα (CC531tFR), were administered i.v. to grow as multifocal syngeneic lung metastases, and rats were administered a rat surrogate for the mouse/human chimeric MOv18 IgE engineered with rat Fc domains and respective effector functions (rat MOv18 IgE). This system permitted targeting of the rat immune system to rat tumour cells by an anti-FRα IgE. Significant efficacy of rat MOv18 IgE in restricting the growth of lung metastases was observed at doses of 5 mg/kg and higher when the antibody was administered fortnightly, compared with controls [186]. The efficacy of rat MOv18 IgE and the equivalent rat IgG2b was then compared: at a 10 mg/kg fortnightly dose, rat MOv18 IgE was significantly superior at restricting tumour growth (Figure 11c).

Overall, in three models of cancer including a patient-derived xenograft and an immunocompetent syngeneic model, the anti-tumour efficacy of MOv18 IgE was reliant on the presence of both an effector cell population and tumour antigen specificity. Furthermore, anti-tumour IgE was more effective than the corresponding IgG.

### 8.3. Evidence for IgE Activating Monocytes and Macrophages against Cancer

#### 8.3.1. Monocytes and Macrophages as Key Effector Cells in MOv18 IgE-Potentiated Anti-Tumour Functions

The mechanisms by which IgE antibodies can exert their anti-tumour effects have been studied and several pieces of evidence support a role for monocytes and macrophages as key effector cells.

*In vitro evidence for monocyte-mediated effector functions:* Monocytes mediate MOv18 IgE-dependent tumour cell killing in vitro by two pathways, ADCC and ADCP, acting through FcεRI and CD23 respectively. FcεRI-expressing primary monocytes principally exert ADCC. MOv18 IgE-potentiated ADCC by monocytes could be blocked with recombinant sFcεRIα [180,181,187], but monocytes could kill tumour cells by ADCP, a function mediated by CD23. MOv18 IgE antibodes can thus engage both receptors to activate effector cells against tumour cells in vitro and in vivo.

*Evidence of macrophage involvement in IgE functions in mouse models:* Pre-clinical in vivo studies in a PDX model suggested that monocytes and macrophages may be important IgE receptor-expressing effector cells that mediate enhanced survival of tumour-bearing mice treated with MOv18 IgE and human PBMCs. Treatment with MOv18 IgE was associated with the histological evidence of tumour infiltration by CD68+ human monocyte-derived macrophages [180,181], suggesting that these were recruited as a part of IgE-mediated anti-tumour functions. Human macrophages were concentrated in stromal areas adjacent to tumour cell islands, while mouse monocytes were abundant in all xenografts examined, irrespective of treatment. In MOv18 IgE-treated mice, human CD68+ macrophage infiltration correlated with longer survival [186]. In the same PDX model, removal of monocytes from the PBMC effector cells abolished the anti-tumour activity of co-administered PBMCs and MOv18 IgE [181]. Reconstitution of monocyte-depleted PBMCs with purified monocytes at proportions equivalent to those in unfractionated PBMCs restored the ability of PBMCs and MOv18 IgE to increase survival to levels equivalent to those seen in mice given whole PBMCs and MOv18 IgE. This survival was significantly longer than monocyte-reconstituted PBMCs alone, or depleted PBMCs with and without MOv18 IgE.

*In vivo evidence of IgE-mediated macrophage activation in a surrogate rat model:* The mechanisms of the action of rat MOv18 IgE in the WAG rat model were examined. Haematoxylin and eosin-stained tumours from different treatment groups in the WAG rat studies revealed more prominent loss of viability, density and demarcation of the tumour areas in rat MOv18 IgE-treated tumours compared to those from animals treated with rat MOv18 IgG2b or a buffer alone. Rat MOv18 IgE-treated tumours demonstrated evidence of considerable necrotic tissue surrounding the smaller tumour cell populations, consistent with previously reported tumour necrosis observed in human xenografts. Inflammatory cells infiltrating between the islands of tumour cells were considerably more pronounced in the rat MOv18 IgE-treated tumours [186].

The density and location of tumour-associated rat CD68+ macrophages in tumours from rats treated with vehicle control, rat MOv18 IgG and rat MOv18 IgE were studied by IHC and flow cytometric analyses of freshly isolated tumour-bearing lung tissues. CD68+ rat macrophages were detected in the TME from all treatment groups by IHC evaluations. Flow cytometric analyses also revealed that the percentage of CD68+ rat macrophages within the tumour-infiltrating CD45+ leukocyte population was higher in the rat MOv18 IgE-treated cohort compared to the rat MOv18 IgG2b-treated or the vehicle alone-treated cohorts. Systemic rat MOv18 IgE treatment was associated with macrophage infiltration deep into the tumour islets. By contrast, macrophages were largely absent from these areas in animals that were administered vehicle alone, or rat MOv18 IgG. The ratio of CD68+ cells within the tumour cell islets compared wth the tumour periphery was greater in the animals administered rat MOv18 IgE than in those with rat MOv18 IgG or vehicle alone, and macrophage infiltration was inversely proportional to tumour occupancy in rats treated with antibodies.

Together, these findings suggest that monocytes and macrophages may be mobilised towards tumours and play crucial roles in the tumour-restricting functions of MOv18 IgE.

#### 8.3.2. Anti-Tumour IgE Directs Monocytes and Macrophages

The TME may influence the immune system to promote either anti-tumour immunity or tumour progression. Tumour associated macrophages (TAMs), characterised by the immune-activating classically-activated (M1) and the tolerance-inducing alternatively activated (M2) extreme phenotypes, are known to suppress or promote the growth of various malignant cells, depending on the biological context [188,189,190]. The activation state of macrophages induced to influx into tumours after administration of rat MOv18 IgE was investigated.

Tumour-infiltrating macrophages from rats treated with rat MOv18 IgE demonstrated an enhanced expression of the M1 co-stimulatory mature APC marker CD80, compared with those from MOv18 IgG2b or buffer-treated groups [186]. However, there was no difference in expression of the M2 marker CD163 between treatment groups. Furthermore, a considerably higher proportion of freshly-isolated CD68+ macrophages from dispersed rat lung tumours of rats administered rat MOv18 IgE were found to express intracellular TNFα, an M1 macrophage marker, compared to MOv18 IgG2b and vehicle-treated tumours. In addition, a higher proportion of CD68+ macrophages from rat MOv18 IgE-treated tumours expressed intracellular IL-10, considered an M2 marker, compared with rat MOv18 IgG2b- and vehicle-treated groups, although this represented a smaller subset compared with the TNFα+ population, with a proportion of cells demonstrating double positivity (TNFα+/IL-10+) within the rat MOv18 IgE-treated cohort. Additional analyses showed significantly elevated circulating TNFα in IgE-treated rat sera compared with controls [191]. The tumour-infiltrating macrophages in rat MOv18 IgE-treated tumours may therefore not be typically M1 or M2, and could instead represent a unique cell subset. Cytokine profile analyses of rat lung (broncho-alveolar lavage, BAL) fluids revealed four analytes, IL-10, TNFα, MCP-1 and IL-1α elevated in the rat MOv18 IgE-treated compared with the rat MOv18 IgG2b-treated cohort [186]. Together with increased levels of macrophage intracellular TNFα and IL-10 detected in the rat MOv18 IgE-treated rats, these data therefore indicate possible roles for TNFα, MCP-1 and IL-10 in the anti-tumoural functions observed following treatment with rat MOv18 IgE. Additional transcriptomic analyses demonstrated the enrichment of gene signatures associated with immune activation pathways, including those associated with IL-12 and Natural Killer (NK) cell-signalling in lungs from rats treated with IgE [191].

Taken together, these data suggest that MOv18 IgE may support TAM populations with mature phenotypes and hybrid M1/M2 features that are able to enter the tumour, trigger sustained immune activating pathways and secretion of IL-10, TNFα, MCP-1 and IL-1α in tumour-bearing lungs.

#### 8.3.3. TNFα/MCP-1 Axis as a Mechanism of MOv18 IgE-Mediated Activation of Human Monocytes

**Figure 12:**
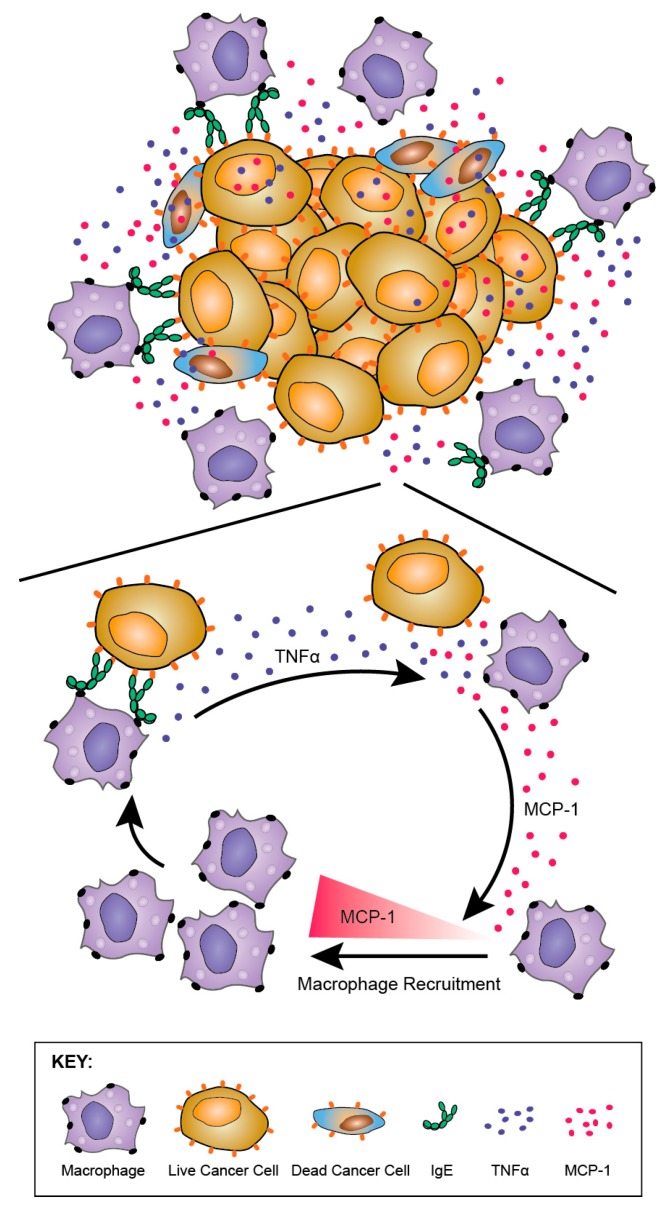
TNFα/MCP-1 cascade as a mechanism of MOv18 IgE functions in vivo. Activation of monocytes/macrophages by MOv18 IgE mediates a TNFα/MCP-1 axis. Cross-linking of IgE upregulates monocyte/macrophage TNFα. TNFα in turn promotes the release of the chemoattractant MCP-1 by monocytes/macrophages and tumour cells in the TME, which could promote potent chemotaxis of further monocytes/macrophages into tumors, resulting in enhanced tumor cell–effector cell interactions and subsequent tumor cell death. Figure adapted by permission from the American Association for Cancer Research. (Josephs, D.H. et al. Anti-Folate Receptor-α IgE but not IgG Recruits Macrophages to Attack Tumors via TNFα/MCP-1 Signaling. *Cancer Res*. 2017, *77*, 1127–1141 [186]).

The potential of, and mechanisms by which, human IgE activates human monocytes was evaluated [186]. Consistent with in vivo findings in the rat model, tumour cell cytotoxicity potentiated by mouse/human chimeric MOv18 IgE and human PBMC effector cells was associated with significantly elevated secreted mediators MCP-1, IL-10, and TNFα in co-culture supernatants, compared with either non-specific NIP IgE-treated or no antibody controls. Cross-linking of IgE, but not IgG, of different antigen specificities on the surface of human monocytes was responsible for the upregulation of TNFα. Cross-linking of IgE bound to tumour cells via the Fab region did not trigger TNFα. Blocking of TNFα receptor reduced IgE-mediated tumour cell cytotoxicity. Together, these findings point to a role for TNFα on IgE-mediated anti-tumour functions. Furthermore, TNFα upregulation by monocytes could in turn promote the release of the monocyte and macrophage chemoattractant MCP-1 by monocytes and a range of tumour cell types. This TNFα/MCP-1 cascade is consistent with the infiltration of macrophages into tumours in at least two in vivo models of cancer, and may point to IgE-mediated mobilisation and activation of monocytes/macrophages into tumours by promoting TNFα-induced production of MCP-1 in the TME (Figure 12).

Together, these findings also draw parallels with increased expression of TNFα, MCP-1 and IL-10 that are reported to be associated with IgE-dependent macrophage-mediated immune responses and clearance of parasites [122,192]. It was originally hypothesised that IgE could mount an allergic response mechanism against cancer. Nonetheless, the lack of IL-4 upregulation, a classic allergic mediator, and the potentiation of a TNFα/MCP-1 axis observed with anti-tumour IgE effector functions, may point to a less dominant role for an allergic, and a more prominent IgE-driven anti-tumour mechanism normally preserved for immune defence and parasite destruction by mobilising and activating macrophages. The implications of these findings may include the re-direction of otherwise inert macrophage populations into tumour lesions, and the activation of IgE-mediated anti-parasitic functions in the Th2-biased TME against tumours [193].

## 9. Towards Clinical Translation of First-In-Class IgE to a First-In-Man Clinical Trial

### 9.1. Predicting Safety of IgE: Using Ex Vivo Functional Assays Adapted from Allergy Diagnosis

In sensitized individuals, minute allergen exposure can trigger life-threatening type I systemic hypersensitivity reactions. Despite preclinical evidence that IgE could have superior efficacy compared with IgG, concerns remain that exogenously administered IgE could trigger a type I hypersensitivity reaction leading to anaphylaxis. For this to occur, monoclonal IgE antibodies bound to FcεRI on effector cells must be cross-linked by soluble multivalent allergen in the circulation [194,195]. Potent allergens can achieve this through forming soluble multimers as discussed above, or by aggregating into complexes cross-linked by polyclonal antibodies, likely to be IgE, specific for these antigens [196,197].

In the context of cancer, it is hypothesised that for an anti-tumour IgE to avoid triggering type I hypersensitivity, the target antigen should be found at low density, and in monomeric form, on healthy cells (and in the circulation) and/or should have only a single IgE-binding epitope, so that IgE cross-linking on the surface of effector cells or bridging with a target cell cannot be achieved [198]. In contrast, for an anti-tumour IgE to have anti-tumour effects, the tumour antigen should be overexpressed on the cancer cells in tissues so that they are densely packed on the cell membrane or in lipid rafts, so that IgE bridging may occur at tumour sites. Tumour-associated antigens such as FRα fulfil these criteria.

To investigate this hypothesis, the ability of MOv18 IgE to trigger basophil degranulation was examined using RBL SX-38 cells engineered to overexpress human FcεRI [182]. Exposure of cells to MOv18 IgE alone did not induce significant degranulation, however the cross-linking of MOv18 IgE bound to the effector cell surface using a polyclonal anti-IgE antibody, or by cross-linking FRα-bound IgE using an anti-FRα polyclonal antibody to mimic the effect of a circulating multimeric antigen, induced appreciable degranulation. In contrast, when cells were incubated with MOv18 IgE and increasing concentrations of recombinant (monovalent) FRα alone, at levels up to 400-fold higher than those reported in ovarian cancer-patient blood, only background levels of degranulation were observed. This was to be expected, since monovalent antigen is generally unable to cross-link FcεRI-bound IgE [182,199]. Furthermore, while naturally shed FRα levels in patient circulation were significantly elevated, compared with those measured from healthy controls, sera from 32 patients with stage III or IV ovarian carcinoma, and from 14 healthy volunteers, induced only background levels of degranulation.

The possibility that circulating tumour cells (CTCs) or tumour cell fragments bearing multiple copies of the target antigen could trigger degranulation was also explored by exposing RBL SX-38 effector cells to MOv18 IgE and serially increasing the number of FRα-expressing IGROV1 ovarian carcinoma cells. Degranulation was only detected at higher E:T cell ratios, well above those recorded in patient blood [182]. This suggests that MOv18 IgE is unlikely to activate effector cells in the presence of even the highest reported concentration of FRα-expressing CTCs. Tumour cells that did not express FRα did not induce degranulation, suggesting that the phenomenon is antigen-specific.

The ability of MOv18 IgE to activate blood basophils ex vivo in fresh unfractionated blood from patients with an ovarian carcinoma was investigated using the basophil activation assay (BAT). BAT is an increasingly useful assay conducted in unfractionated blood for detecting the propensity for type I hypersensitivity to a large range of allergens [200,201,202,203], including medicinal drugs and those used in oncology. It is designed to measure elevated cell surface CD63 expression within 10-15 min of stimulation as an early sign of type I hypersensitivity, which precedes degranulation [204]. MOv18 IgE at a range of concentrations had no effect on the level of CD63 expression in whole blood samples from healthy volunteers or from patients with an ovarian carcinoma, despite detectable circulating concentrations of FRα in the blood of some of these patients. Furthermore, MOv18 IgE with the addition of exogenous soluble FRα, even at concentrations 10-fold higher than those observed in patients, did not increase CD63 expression by human basophils. In contrast, cross-linking of effector cell FcεRI using either an anti-FcεRI or anti-IgE polyclonal antibody clearly augmented CD63 expression [182]. MOv18 IgE was therefore unable to produce significant basophil activation in human blood specimens.

In the same study, sera from 24 patients with detectable levels of circulating FRα antigen were also screened for the presence of anti-FRα IgG auto-antibodies. Such antibodies might potentially cross-link the soluble FRα bound to MOv18 IgE on the surface of basophils. In 6 of 24 patient sera, IgG auto-antibodies were detected in the range of 3–43 ng/mL. However, when tested in the RBL SX-38 degranulation assay, sera from these patients did not trigger any functional degranulation in the presence of MOv18 IgE. Sera from two patients were also studied in the BAT assay and induced with no increase in CD63 expression by the patients’ blood basophils [182].

In conclusion, no evidence of effector cell activation or degranulation could be detected in validated models of allergy using recombinant FRα or patient blood and sera. In addition, no degranulation was mediated by MOv18 IgE at worst case physiological blood CTC-to-effector cell ratios or by patient anti-FRα IgG auto-antibodies. Overall, these data indicate that when ovarian carcinoma patients are treated with MOv18 IgE, FcεRI-mediated activation of effector cells may potentially occur within the tumour mass, but is less likely in the circulation.

### 9.2. Predicting Safety of IgE: In Vivo Models

Selection of preclinical models to help predict the safety of IgE antibody immunotherapy of cancer is still in its very early stages, and pharmacologically relevant species are being sought. An anti-human HER2/*neu* IgE was well-tolerated when introduced to cynomolgus monkeys [160]. Cross-species reactivity of mouse/human chimeric MOv18 IgE was demonstrated in cynomolgus monkey immune effector cells [205]. However, the kinetics of MOv18 IgE interaction with effector cells, and the phenotype of the activated effector cells, differed between the two species; human IgE featured a faster dissociation from cynomolgus monkey effector cells, compared with human immune effector cells. Human IgE triggered different cytokine release profiles by human and cynomolgus monkey immune effector cells. Therefore, the extrapolation of cynomolgus data to humans may be unreliable [205].

For these reasons, a surrogate syngeneic tumour model in immunocompetent (WAG) rats (discussed above) was designed to evaluate the safety profile of anti-tumour IgE. This species was selected because the IgE system of the rat bears many similarities to that of a human, and the use of the rat MOv18 IgE in the WAG rat would allow the characterisation of IgE-mediated responses that would not be possible in healthy primate models.

**Figure 13:**
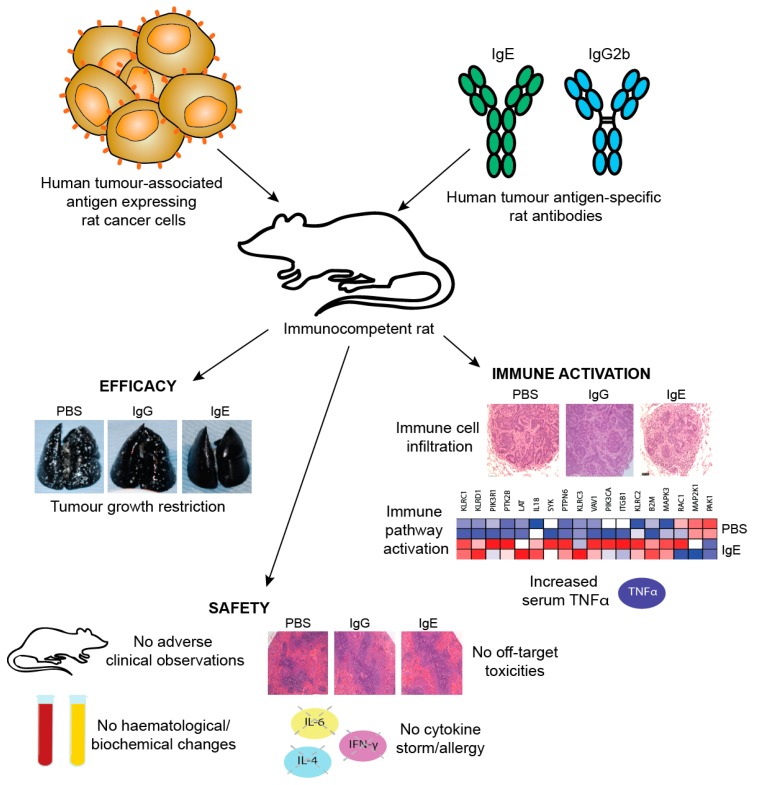
In vivo safety evaluations of MOv18 IgE. A surrogate syngeneic tumour model in immunocompetent WAG rats was designed to evaluate the safety profile of MOv18 IgE. Rat CC531 colon adenocarcinoma cells, engineered to express the human FRα, were administered i.v. to grow as lung metastases and animals were treated with either rat MOv18 IgE or the IgG2b equivalent. This model demonstrated the superior efficacy of IgE compared with the IgG counterpart (representative images of Indian ink-stained lungs shown). Efficacy was observed in the absence of any adverse clinical observations, off-target toxicities (H&E-stained spleen shown), or haematological or biochemical changes. Furthermore, no evidence of cytokine storm (lack of IL-6 or IFNγ upregulation) or allergic response (lack of IL-4 upregulation) was detected. In the same model, MOv18 IgE treatment was associated with the restriction of tumour growth, alongside enhanced immune cell infiltration in tumours (H&E-stained lung shown) and elevated immunological pathway activation gene signatures. Additionally, increased tumour and serum TNFα were measured in association with IgE treatment. Figure adapted by permission from John Wiley & Sons, Inc. (Josephs, D.H. et al. An immunologically relevant rodent model demonstrates safety of therapy using a tumour-specific IgE. *Allergy* 2018, *73*, 2328–2341 [191]).

Preclinical efficacy studies using tumour-bearing rats showed restriction of tumour growth in the absence of any evidence of acute toxicity with rat MOv18 IgE (or with the equivalent rat MOv18 IgG2b), despite the natural presence of IgE effector cells capable of IgE-mediated degranulation such as basophils and mast cells in this species. No evidence of a cytokine storm (lack of IL-6 or IFNγ) or signals of an allergic response (IL-4) were detected, while elevated immunological pathway activation gene signatures, tumour and serum TNFα elevation and enhanced macrophage infiltration into tumours, thought to be associated with anti-tumoral efficacy, were associated with IgE treatment (Figure 13) [191].

In concordance, in previous immunodeficient mouse models of human FRα-expressing carcinoma xenografts, the administration of mouse/human chimeric MOv18 IgE or MOv18 IgG1 together with human peripheral blood lymphocytes and peripheral blood mononuclear cells did not trigger any toxic effects, despite the presence of human basophils and eosinophils, including those from allergic human donors [147,179,181], in these effector cell preparations. Further support for this concept comes from published data demonstrating the induction of IgE through tumour antigen mimotope vaccination, detected in the absence of any toxicities or signs of type I hypersensitivity [206]. Furthermore, IgE specific to tumour antigens and with tumoricidal properties has been reported in patients with head and neck cancer and pancreatic cancer, in the circulation and tumour tissues [104,105], without anaphylaxis occurring.

Finally, dogs may be an alternative model to examine the safety and anti-tumour functions of IgE, since this species is known for susceptibility to both cancer, including spontaneous mammary carcinomas, and allergy, with strong similarities of FcεR expression and distribution on immune cells compared with humans [207,208,209]. Efforts are underway to design canine versions of anti-tumour IgEs with a view to conduct safety and efficacy studies [152].

### 9.3. Monitoring Antibody Safety in Trials

Translation to clinical testing is expected to entail careful monitoring of patients and measuring functional readouts and immunological markers of type I hypersensitivity following administration of MOv18 IgE due to the potential for basophil and/or mast cell degranulation. Functional tests may monitor the propensity to trigger basophil activation and mast cell degranulation in patient blood and sera ex vivo, all measured at different points of drug administration. Monitoring would include clinical signs of type I hypersensitivity, changes in serum levels of β-tryptase, total and tumour antigen-specific IgE, circulating tumour antigen and autoantibodies to the target antigen. Specifically, serum β-tryptase elevation signifying mast cell degranulation during clinical testing may be important to help distinguish cytokine release-type infusion reactions from type I hypersensitivity [210,211].

## 10. Thoughts for the Design of New IgE-Based Therapeutic Agents

### 10.1. Expression Systems and IgE Glyco-Profiling

Production of IgE for clinical study requires the development of GMP processes that ensure swift production of an antibody with sufficient quality, purity and stability profiles. Importantly, the product must show physiochemical and functional profiles compatible with those of the laboratory grade material. Additionally, IgE antibodies display seven glycosylation sites, six of which comprise complex N-glycans, potentially with terminal galactose, fucose and sialic acid residues, as discussed above (and illustrated for IgG in Figure 1c). Due to its heavily glycosylated structure, the glycosylation profile of IgE antibodies must also be considered with regard to achieving a consistent antibody structural and functional product profile for clinical application. Carbohydrates may influence the affinity for the target antigen, biodistribution, effector cell trafficking to tissues and antibody pharmacokinetics; the high-mannonse structure at Asn394 (Figure 1d) may, as we have discussed, have functional significance [41,50]. Monitoring the structural and functional integrity of IgE is therefore warranted at all stages of research, development and manufacturing for pre-clinical and clinical evaluations. Furthermore, the nature of the expression system may impact the glycosylation profile and must be carefully considered when designing an IgE class therapeutic agent [153]. For instance, the carbohydrate profile of IgE antibodies produced using a human expression system, may differ from that of plant-expressed IgE [150]. Further study of glycan content will undoubtedly provide important information for further understanding structure-function relationships in IgE.

### 10.2. Selecting Tumour Targets and Malignant Indications for IgE Therapeutic Agents

Rational design of suitable therapeutic agents should aim to take advantage of the tissue-resident immune surveillance exerted by IgE antibodies that can be directed against cancer antigens, whilst minimising the risk of the potential toxic effects of the therapeutic agent. Malignant indications could be selected according to whether tumour cells are likely to reside in tissues in which important IgE effector cells such as macrophages are also found. Indications in which tumour cells and tumour cell fragments do not circulate would be preferable, since following systemic administration of anti-tumour IgE, basophils loaded with anti-tumour IgE could encounter circulating cancer cells bearing multiple copies of the target antigen; such interactions might trigger degranulation and potential type I hypersensitivity. Important criteria for the selection of cancer antigen targets would include high expression on the tumour with minimal and restricted distribution in normal tissues away from patient circulation. Furthermore, selection of single epitopes on tumour antigens and antigens that do not shed in multimeric forms in patient circulation would be key criteria for target selection.

### 10.3. Challenges for IgE-Based Therapies

Within the fields of Immunology, Allergy and AllergoOncology, there are many aspects of IgE biology that are yet to be explored. The most prominent unknowns in the field are: defining the dynamics of antibody trafficking to tumours, recruiting monocytes into tumour lesions and engaging local tumour-associated macrophages; pharmacokinetics in patient circulation and biodistribution in health and disease settings; the roles and anti-tumour functions of mast cells; unexplored mechanisms of action beyond the TNFα/MCP-1 cascade; the existence of modulatory mechanisms for IgE despite the lack of any known inhibitory FcεR; the impact of target antigen expression levels and distribution in tumour lesions on the anti-tumour efficacy of IgE antibodies; stratification of patients with tumours featuring immune tumour environments congruent to IgE antibody therapy; the most suitable administration route, and malignant indication to help refine treatment and maximise patient benefit.

Evidence from a number of studies points to monocytes and macrophages as important effector cells that participate in the anti-tumour functions of IgE in vitro and in vivo [193]. On the other hand, mast cells express far higher levels of FcεRI compared with monocytes and macrophages, and constitute another key effector cell population that may contribute to the cancer growth-restricting functions of anti-tumour IgE antibodies. Mast cells can be activated upon crosslinking of FcεRI by IgE in the presence of multivalent antigens, to degranulate and release toxic mediators in tissues such as the skin and gut. These functions of mast cells have been known to be directed to destroy parasites [5,97]. The significance of mast cell infiltration in tumour lesions has been controversial [212], however there have been reports of associations with more favourable clinical outcomes [213]. Tumour- and tissue-resident mast cells may also contribute to IgE-mediated enhanced TNFα expression and heightened immune responses in the TME [214]. Mast cells could be recruited towards tumour lesions either through tumour cell-produced MCP-1 [215], and more prominently through the anti-tumour IgE-potentiated TNFα/MCP-1 axis discussed above [186,191]. However, the roles of mast cells in the context of anti-tumour IgE mechanisms of action and efficacy require further study.

Further areas for investigation include the impact of clinically available therapies such as chemotherapies, checkpoint inhibitors, steroids, and targeted treatments on the following: effector cells and IgE therapeutic efficacy and safety; expression of IgE Fc receptors by immune cells in different cancer types and patient tumours; mechanisms by which IgE acts on the TME, including IgE receptor-expressing and non-expressing cells, and their recruitment into tumours.

A number of antibodies engineered with IgE Fc regions have been shown to engender potent effector functions and restrict tumour growth in disparate model systems. These include antibodies recognizing epitopes found on clinically validated tumour targets such as HER2/*neu*. It is to be hoped that IgE antibodies against these targets will progress along the translational pipeline towards clinical testing. The field of AllergoOncology, including the use of IgE antibodies for cancer treatment, will undoubtedly enrich our understanding of human immunity and responses in health and malignant disease, and both inform and transform the design of future immunotherapeutic agents.

## Abbreviations

ADCC	antibody-dependent cell-mediated cytotoxicity
ADCP	antibody-dependent cell-mediated phagocytosis
APC	antigen presenting cell
BAL	broncho-alveolar lavage
BAT	basophil activation test
CCA	colorectal cancer antigen
CDR	complementarity-determining region
CTCs	circulating tumour cells
CTL	cytotoxic T lymphocyte
DCs	dendritic cells
EGFR	epidermal growth factor receptor
EM	electron microscopy
FR	framework region
FRα	folate receptor alpha
FRET	fluorescence (Förster) resonance energy transfer
GMP	Good Manufacturing Practice
IHC	immunohistochemical/immunohistochemistry
i.p.	intraperitoneal
i.v.	intravenous
MCP-1	macrophage chemoattractant protein-1
MD	molecular dynamics
MMTV	mammary tumour virus
NIP	4-hydroxy-3-nitro-phenacetyl
NK	Natural Killer
PBMCs	peripheral blood mononuclear cells
PDX	patient-derived xenograft
PIPE	Polymerase Incomplete Primer Extension
PSA	prostate specific antigen
RBL	rat basophil leukaemia
SAXS	small-angle X-ray scattering
s.c.	subcutaneous
Th	T helper
TME	tumour microenvironment
TNFα	tumour necrosis factor
UCOE	Ubiquitous Chromatin Opening Elements
WAG	Wistar Albino Glaxo
